# Supplementary material for: PRRX1 is a master transcription factor of stromal fibroblasts for myofibroblastic lineage progression
Source: Nat Commun. 2022 May 19;13:2793. doi: 10.1038/s41467-022-30484-4 (PMC9120014; doi:10.1038/s41467-022-30484-4)
Supplement: Supplementary file 1 — Supplementary information [file 41467_2022_30484_MOESM1_ESM.pdf]

Supplementary Information

PRRX1 is a master transcription factor of stromal fibroblasts for myofibroblastic lineage progression

Keun-Woo Lee, So-Young Yeo, Jeong-Ryeol Gong, Ok-Jae Koo, Insuk Sohn, Woo Yong Lee, Hee Cheol Kim, Seong Hyeon Yun, Yong Beom Cho, Mi-Ae Choi, Sugyun An, Juhee Kim, Chang Ohk Sung, Kwang-Hyun Cho, and Seok-Hyung Kim

Contents

1. Supplementary Table 1 -- 2
2. Supplementary Methods --- 7
3. Supplementary Figures -- 12
4. Supplementary References -- 52

Supplementary Table 1. Key resources table.

REAGENT or RESOURCE	SOURCE	IDENTIFIER
Antibodies		
Anti-Prrx1	Origene	Cat# TA803116,RRID:AB_2620166
Anti-Prrx1	LS-bio	Cat# LS-C336798,RRID: AB_2725124
Anti-aSMA	DAKO	Cat# M0851; RRID:AB_2313736
Anti-PCNA	Abcam	Cat# ab29, RRID:AB_303394
Anti-Tenascin C	Abcam	Cat# ab108930, RRID:AB_10865908
Anti-PDGFRa	Cell signaling	Cat# 3164, RRID:AB_2162351
Anti-H3K27ac	Abcam	Cat# ab4729, RRID:AB_2118291
Anti-Histone H3 (tri methyl K4)	Abcam	Cat# ab8580, RRID:AB_306649
Anti- Cas9 (7A9-3A3)	Cell signaling	Cat# 14697S, RRID:AB_2750916
Anti- F4/80	Cell signaling	Cat# 70076S, RRID:AB_2799771
Anti-normal mouse IgG	Santa Cruz Biotechnology	Cat# sc-2025, RRID:AB_737182
Bacterial and Virus Strains		
pLXSN16E6E7	Addgene	Cat# 52394 RRID:Addgene_52394
Biological Samples		
Human stomach/colon biopsies	Samsung medical center	N/A
Chemicals, Peptides, and Recombinant Proteins		
Recombinant Mouse TGF-β1	Cell signaling	Cat# 5231
Recombinant Mouse Sonic Hedgehog/Shh	R&D Systems	Cat# 464-SH-025/CF
cis-Diammineplatinum(II) dichloride	Sigma-Aldrich	Cat# P4394
D-Luciferin Firefly	Biosynth	Cat# J-012343
Azoxymethane	Sigma-Aldrich	Cat# A5486
Dextran sulfate sodium salt	MP Biomedicals	Cat# 02101516-CF
Critical Commercial Assays		
Cytokine array kit	R&D Systems	Cat# ARY005B
Cell contraction assay kit	Cell Biolabs	Cat# CBA-201
BCA Protein Assay kit	Thermo Fisher	Cat# 23225
Anti-mouse/rabbit polymer kit	DAKO	Cat# K5007
ImmPACT AEC Peroxidase Substrate	Vector Labs	Cat# sk-4205
Annexin – V APC apoptosis detection Kit	BD Biosciences	Cat# 550474
Deposited Data		
Three types of murine fibroblasts RNA-seq	This paper	GSE169703
Three types of murine fibroblasts ChIP-seq	This paper	GSE169723
RNA-seq data of Co-cultures of cancer spheroid with fibroblasts	This paper	GSE169697
Human cancer associated fibroblasts RNA-seq	This paper	GSE169720

Human cancer associated fibroblasts ChIP-seq	This paper	GSE169601
Single cell RNA-seq from murine wound tissue	This paper	GSE169704
Colorectal cancer single cell RNA-seq	Lee *et. al.*[1]	GSE132465, GSE144735
Lung cancer single cell RNA-seq	Lambrechts *et. al.*[2]	E-MTAB-6149, E-MTAB-6653
Ovarian cancer single cell RNA-seq	Qian *et. al.*[3]	E-MTAB-8107
Squamous cell carcinoma single cell RNA-seq	Ji *et. al.*[4]	GSE144240
Stomach cancer single cell RNA-seq	Sathe *et. al.*[5]	phs001818.v1.p.1.
Pancreatic cancer single cell RNA-seq	Peng *et. al.*[6]	GSA: CRA001160
HNSCC single cell RNA-seq	Puram *et. al.*[7]	GSE103322
Mouse embryonic like cell single cell RNA-seq	Zhao *et. al.*[8]	GSE114952
Mouse skin wound single cell RNA-seq	Guerrero-Juarez *et. al.*[9]	GSE113854
Mouse pancreatic CAF single cell RNA-seq	Hosein *et. al.*[10]	GSE125588
Experimental Models: Cell Lines		
MMTV-PyMT derived primary fibroblasts	Isolated in this study (FVB/N)	N/A
Mouse embryonic fibroblasts	Isolated in study (C57BL/6)	N/A
Mouse dermal fibroblasts	Isolated in study (C57BL/6)	N/A
Human : HT29	KCLB	Cat# 30038, RRID:CVCL_0320
Human : HCT116	KCLB	Cat# 10247, RRID:CVCL_0291
Human : MKN28	KCLB	Cat# 80102, RRID:CVCL_1416
Human : SNF32	Yeo *et. al.*[11]	N/A
Human : Colon CAFs (1-9)	Isolated in this study	N/A
Mouse : LLC1 (modified to express GFP and Luciferase)	ATCC	Cat# CRL-1642, RRID:CVCL_4358
Mouse : 168FARN (modified to express GFP)	Jing Yang Laboratory	RRID:CVCL_0I86
Mouse : MC38 (modified to express GFP and Luciferase)	Choy Laboratory	RRID:CVCL_B288
Human : SNU668	KCLB	Cat# 00668, RRID:CVCL_5081
Experimental Models: Organisms/Strains		
Mouse: MMTV-PyMT (backcrossed to FVB/N)	Jackson Laboratory	RRID:IMSR_GPT:T004993
Mouse: Prrx1tm1Jfm/Mmmh (backcrossed to C57BL6)	MMRRC	RRID:MMRRC_000347-MU
Mouse: S100a4-cre (backcrossed to BALB/c)	Jackson Laboratory	IMSR Cat# JAX:012641, RRID:IMSR_JAX:012641
Mouse: Rosa26-LSL-Cas9 knockin (backcrossed to C57BL6)	Jackson Laboratory	RRID:IMSR_JAX:024857
Mouse: C57BL/6J	Jackson Laboratory	RRID:IMSR_JAX:000664

Mouse: NSG (NOD.Cg-Prkdcscid Il2rgtm1Wjl/SzJ)	Jackson Laboratory	RRID:IMSR_NM-NSG-012 Stock No: 005557
Mouse: NOD/SCID	Jackson Laboratory	RRID:IMSR_JAX:001303
Mouse: Col1a2-rtTA (backcrossed to C57BL6)	This paper	N/A
Mouse: TetO7-Prrx1-Luciferase (backcrossed to C57BL6)	This paper	N/A
Oligonucleotides		
shRNA targeting Prrx1-1 5'CCGGGTCCCTCCCAAGATGTTGTTTCTCGAGAA ACAACATCTTGGGAGGGACTTTTTG 3' Forward	This paper	N/A
shRNA targeting Prrx1-2 5'CCGGGCAGGCTTTGGAGCGTGTCTTCTCGAGAA GACACGCTCCAAAGCCTGCTTTTTG 3' Forward	This paper	N/A
shScrbl control for Prrx1: 5'CCGGCAACAAGATGAAGAGCACCAACTCGAG TTGGTGCTCTTCATCTTGTTGTTTTTG3' Forward	This paper	N/A
Mouse Postn Forward : 5' TGGTATCAAGGTGCTATCTGCG 3' Forward	This paper	N/A
Mouse Postn Reverse : 5' AATGCCCAGCGTGCCATAA 3' Forward	This paper	N/A
Mouse Wnt5a Forward : 5' CAACTGGCAGGACTTTCTCAA 3' Forward	This paper	N/A
Mouse Wnt5a Reverse : 5' CCTTCTCCAATGTACTGCATGTG 3' Forward	This paper	N/A
Mouse Igf1 Forward : 5'CACATCATGTCGTCTTCACACC3' Forward	This paper	N/A
Mouse Igf1 Reverse : 5' GGAAGCAACACTCATCCACAATG3' Forward	This paper	N/A
Mouse Adamts2 Forward : 5'GAGACGTGGCAGACTTACCTA3' Forward	This paper	N/A
Mouse Adamts2 Reverse : 5'CCAGAGGTTCGATAAAGAACTCC3' Forward	This paper	N/A
Mouse Aldh1a3 Forward : 5'ATCAACAACGACTGGCACGAA3' Forward	This paper	N/A
Mouse Aldh1a3 Reverse : 5' CACATCGGGCTTATCTCCTTC3' Forward	This paper	N/A
Mouse Tgfb2 Forward : 5'CTTCGACGTGACAGACGCT3' Forward	This paper	N/A
Mouse Tgfb2 Reverse : 5'GCAGGGGCAGTGTAAACTTATT3' Forward	This paper	N/A
Mouse Cdh1 Forward : 5'CAGTTCCGAGGTCTACACCTT3' Forward	This paper	N/A
Mouse Cdh1 Reverse : 5'TGAATCGGGAGTCTTCCGAAAA3' Forward	This paper	N/A
Mouse Vimentin Forward : 5'CGTCCACACGCACCTACAG3' Forward	This paper	N/A

Mouse Vimentin Reverse : 5'GGGGGATGAGGAATAGAGGCT3' Forward	This paper	N/A
Mouse Tgfb1 Forward : 5'CCACCTGCAAGACCATCGAC3' Forward	This paper	N/A
Mouse Tgfb1 Reverse : 5'CTGGCGAGCCTTAGTTTGGAC3' Forward	This paper	N/A
Mouse Fn1 Forward : 5' ATGTGGACCCCTCCTGATAGT3' Forward	This paper	N/A
Mouse Fn1 Reverse : 5'GCCCAGTGATTTCAGCAAAGG3' Forward	This paper	N/A
Mouse GAPDH Forward : 5' AATGGATTTGGACGCATTGGT3' Forward	This paper	N/A
Mouse GAPDH Reverse : 5' TTTGCACTGGTACGTGTTGAT3' Forward	This paper	N/A
Mouse Prrx1 Forward : 5' GAGCGTGTCTTTGAGCGGA3' Forward	This paper	N/A
Mouse Prrx1 Reverse : 5' CATGTGGCAGAATAAGTAGCCAT3' Forward	This paper	N/A
Recombinant DNA		
pFU-Luc2-GFP	Gambhir Laboratory (Stanford University)	N/A
pHR.CMV.FLAG.IRES.Hygro	Yeo *et. al.*[11]	N/A
pHR.CMV.Prrx1a.IRES.Hygro	Yeo *et. al.*[11]	N/A
pHR.CMV.Prrx1b.IRES.Hygro	Yeo *et. al.*[11]	N/A
pLVeGFP.Basic.puro	Yeo *et. al.*[11]	N/A
pLVeGFP.hPRRX1.puro	This paper	N/A
lentiCRISPRV2_tdTomato	This paper	N/A
lentiCRISPRV2_tdTomato_sgPrrx1	This paper	N/A
Software and Algorithms		
Trimmomatic	Bolger *et. al.*[12]	http://www.usadellab.org/cms/index.php?page=trimmomatic
Bowtie	Langmead *et. al.*[13]	http://bowtie-bio.sourceforge.net/index.shtml
MACS2	Zhang *et. al.*[14]	https://github.com/macs3-project/MACS
ROSE	Whyte *et. al.*[15]	http://younglab.wi.mit.edu/super_enhancer_code.html
Coltron	Lin *et. al.*[16]	https://pypi.org/project/coltron/
Samtools	Li et. al.[17]	https://github.com/samtools/
Bedtools	Quinlan *et. al.*[18]	https://github.com/arq5x/bedtools2
Homer	Heinz *et. al.*[19]	http://homer.ucsd.edu/homer/

Integrative Genomics Viewer	Robinson *et. al.*[20]	http://software.broadinstitute.org/software/igv/
Deeptools	Ramírez *et. al.*[21]	https://github.com/deeptools/deepTools/blob/develop/docs/index.rst
EnrichR	Kuleshov *et. al.*[22]	https://cran.r-project.org/web/packages/enrichR/index.html
DiffBind	Ross-Innes *et. al.*[23]	https://www.bioconductor.org/packages/release/bioc/html/DiffBind.html
STAR	Dobin *et. al.*[24]	https://github.com/alexdobin/STAR
HTSeq	Anders *et. al.*[25]	https://htseq.readthedocs.io/en/master/overview.html
fgsea	Sergushichev *et. al.*[26]	https://bioconductor.org/packages/release/bioc/html/fgsea.html
GSVA	Hänzelmann *et. al.*[27]	https://www.bioconductor.org/packages/release/bioc/html/GSVA.html
Cellranger	Zheng *et. al.*[28]	https://support.10xgenomics.com/single-cell-gene-expression/
Seurat	Hao *et. al.*[29]	https://satijalab.org/seurat/index.html
CellAssigner	Zhang *et. al.*[30]	https://github.com/Irrationone/cellassign
NichenetR	Browaeys *et. al.*[31]	https://github.com/saeyslab/nichenetr
DEseq2	Love *et. al.*[32]	https://bioconductor.org/packages/release/bioc/html/DESeq2.html
R	N/A	https://www.r-project.org/
Reagent or Resource		
Fast sybr green master mix	Applied Biosystems	Cat# 4385617
Rneasy Plus Mini Kit	Qiagen	Cat# 74134
Chromium Single Cell 3’ v2 reagent kit	10x Genomics	PN-120237
NEBNext® UltraTM DNA Library Prep Kit	New England Biolabs	E7370L
TruSeq RNA Sample Preparation kit	Illumina	RS-121-2001

Supplementary methods

Single cell RNA-Seq (scRNA-seq) and Data Analysis

Collect seven single cell RNA seq data from public database

The several scRNA-seq datasets used for this study are published and publicly available. We obtained the human colorectal cancer datasets[1] from Gene Expression Omnibus (GEO) with accession GEO: GSE132465 and GEO: GSE144735. The human lung cancer datasets[2] obtained from ArrayExpress: E-MTAB-6149, E-MTAB-6653. The human ovarian cancer datasets[3] were derived from ArrayExpress: E-MTAB-8107. The human stomach cancer datasets[5] were derived from the database of Genotypes and Phenotypes (dbGaP) with accession dbGAP: phs001818.v1.p.1. The expression data for human skin cancer datasets[4] and human head and neck cancer datasets[7] obtained from GEO with accession GEO: GSE144240 and GEO: GSE103322, respectively. Also, we obtained the human pancreatic cancer datasets[6] from Genome Sequence Archive (GSA) with accession GSA: CRA001160.

Preprocessing scRNA-seq and data

Single cell RNA sequencing (scRNA-seq) was performed from mouse wound tissue after 10days post wound using 10X Chromium 3' v2 chemistry. The raw scRNA-seq reads were aligned to the Mus musculus reference genome, mm10, using cellranger v3.1.0 pipeline from 10X genomics. For the initial quality control, we removed cells with fewer than 200 detected genes and genes expressed in fewer than 3 cells. In addition, we discarded apoptotic cells expressing more than 5% mitochondrial transcripts since they are regarded as low-quality cells. After filtering, 2,519 cells were used for further analysis. For each cell, log-normalization was performed by scaling to a constant total read count per cell (100,000) and log-transformation. For visualization, we performed principal component analysis with 2,000 highly variable genes for initial dimensionality reduction and performed T-distributed stochastic neighbor embedding (tSNE) and Uniform Manifold Approximation and Projection (UMAP) to reduce PCA dimensions into 2D space. All of these aforementioned analyses were performed using Seurat v4.0.0 package in R (v 3.6.3)[33].

Probabilistic single cell annotation for 11 cell types in eight tissue

We use the hierarchical statistical framework CellAssign[30] (version 0.99.16) to calculate the probability that each cell belongs to a cell type. Previously used markers collected in previous studies were used[34]. The following list of genes was used for single cell annotation. BANK1, CD79A, CD79B, FCER2, FCRL2, FCRL5, MS4A1, PAX5, POU2AF1, STAP1 TCL1A (B cell); ADAM33, CLDN11, COL1A1, COL3A1 ,COL14A1 ,CRISPLD2, CXCL14, DPT, F3, FBLN1, ISLR, LUM, MEG3, MFAP5, PRELP, PTGIS, SFRP2, SFRP4, SYNPO2, TMEM119 (Fibroblast); ANKRD55, DGKA, FOXP3, NT4, IL2RA, MDS2, RCAN3, TBC1D4, TRAT1 (CD4 T cell);

CD8B, HAUS3, JAKMIP1, NAA16, TSPYL1 (CD8 T cell); CDH5, CLDN5, CLEC14A, CXorf36, ECSCR, F2RL3, FLT1, FLT4, GPR4, GPR182, KDR, MMRN1, MMRN2, MYCT1, PTPRB, RHOJ, SLCO2A1, SOX18, STAB2, VWF (Endothelial cell); APOC1, C1QC, CD14, CD163, CD300C, CD300E, CSF1R, F13A1, FPR3, HAMP, IL1B, LILRB4, MS4A6A, MSR1, SIGLEC1, VSIG4 (Macrophage); CD33, CD300C, CD300E, CECR1, CLEC6A, CPVL, EGR2, EREG, MS4A6A, NAGA, SLC37A2 (Monocyte); CEACAM3, CNTNAP3, CXCR1, CYP4F3, FFAR2, HIST1H2BC, HIST1H3D, KY, MMP25, PGLYRP1, SLC12A1, TAS2R40 (Neutrophils); CD160, CLIC3, FGFBP2, GNLY, GNPTAB, KLRF1, NCR1, NMUR1, S1PR5, SH2D1B (NK cell); BCL11B, CD5, CD28, IL7R, ITK, THEMIS, UBASH3A (T cell). Next, the cell-specific size factors in the R package were calculated from the formula found by the function found in the scarn package (version 1.12.1). Probability was assigned to each cell type for the annotated entities.

Identifying PRRX1 as the most specific TF for activated CAF of activated CAFs

To find out highly correlated TF with activated CAF, we used the annotated scRNA-seq. First, we identified differentially expressed genes between normal fibroblasts (NFs) and cancer associated fibroblasts (CAFs) using ‘FindAllMarkers’ function with default parameters in Seurat[33]. Second, we selected only the six TFs among common DEGs. Third, Pearson Correlation Coefficient (PCC) between expression level of the six TFs and activated fibroblast scores were calculated. Finally, we selected the TF of the highest PCC.

Preparation of scRNA-seq isolated from mouse wound tissue

Adult wild type mice (Balb/c) were used for these scRNA-seq. 12mm biopsy punch skin wound was placed on the dorsal of mice. After 10 days(at day10 post wound), tissue of wound area were excised. The tissue were cut into small pieces, minced with scalpels and dissociated with DMEM/F-12 serum medium containing collagenase I in a 37'C for 12hrs using an shaker. Cells from wound tissue were transferred to a 50ml tube, and the dead cells were removed using ficoll seperation. For scRNA sequencing and processing, 3' single cell RNA sequencing was performed using single cell A chip kit, Single Cell 3′ Library and Gel Bead Kit V2, and i7 Multiplex Kit (10x Genomics, Pleasanton, CA, USA) following the manufacturer’s user guide. Library was sequenced on the Illumina HiSeq2500 platform

Unsupervised trajectory using single cell RNA-seq using monocle2

We used monocle2[35] to construct single cell trajectories. First, we filtered low-quality genes that are expressed in less than 0.1% cells of the data set. Then we divided the cells into PRRX1-high (+) and low (-) group according to PRRX1 gene expression level using bimodal test and performed differential expression analysis between PRRX1-high (+) and low (-) group. Next, we selected set of genes to order cells based on p-value of differential expression analysis. Finally, we reduced the high-dimensional space into two dimensions using DDRTree algorithm and ordered the cells according to pseudotime. We visualized module scores and gene expression levels in the unsupervised trajectory by mapping colors to each cell according to their values.

Computing module score of specific feature signatures in scRNA-seq data using Seurat's addmodulescore function

We calculated the module scores of single-cell RNA seq data using the 'AddModuleScore' function in Seurat. A module score is the average expression levels of genes associated with a specific feature in a particular single cell minus that of a randomly selected control group. A high module score suggests that genes in a specific feature are expressed more highly in a particular single cell than expected. Using the 'AddModuleScore' function, we calculated fibroblast activation score using gene set associated with fibroblast activation[36], ECM organization score using genes in GO_EXTRACELLULAR_MATRIX, and Myofibroblast like score using genes in GO_REGULATION_OF_SUPRAMOLECULAR_FIBER_ORGANIZATION in the Molecular Signatures Database (MSigDB) [37].

Construction of cell-cell interaction networks between CAFs and cancer cells isolated from eight cancer tissues

To identify activated ligands between sender cells (High PRRX1-expressing and low PRRX1-expressing CAFs) and receiver cells (various cancer cells), NicheNet was employed to predict the activated ligands from the scRNA-seq data. To identify only activated ligands, NicheNet uses the knowledge-based prior model of potential ligand-target regulatory networks. It then predicts the influence of each ligand over the expression of receiver cells. To run NicheNet, DEGs between activated cells and inactivated cells in receiver cell types (various cancer cells isolated eight cancer tissues) were screened first using the FindMarker function in Seurat with default parameters. The predict_ ligand_activities function in NicheNet was then used with the identified DEGs to predict the ligands bound to receiver cells.

Copy number analysis of sc-RNA-seq data

To identify aneuploid and diploid cells, we used the CopyKAT algorithm to calculate DNA copy number alteration from scRNA-seq data[38].

Bulk RNA Sequencing and Data Analysis

Library preparation

Sequencing libraries were prepared using TruSeq RNA Sample Preparation kit v2 (Illumina). After pooled libraries were denatured, sequencing of each library was carried out using the 100 bp paired-end mode of the TruSeq Rapid PE Cluster Kit and TruSeq Rapid SBS Kit with HiSeq 2500 (Illumina Technologies., USA).

Alignment and pre-processing of Bulk RNA-seq data

To quantify total RNA of murine fibroblasts and human CAFs, we prepared Sequencing libraries using TruSeq RNA Sample Preparation kit v2 (Illumina., USA). After pooled libraries

were denatured, sequencing of each library was carried out using the 100 bp paired-end mode of the TruSeq Rapid PE Cluster Kit and TruSeq Rapid SBS Kit with HiSeq 2500 (Illumina Technologies., USA). The prepared RNA-seq data were trimmed using Trimmomatic version 0.39[12]. The trimmed reads were aligned to the mm10 reference genome using STAR version 2.7.7a[24] with the default parameter. The mapped reads were indexed and sorted by samtools version 1.7[17]. Then HTSeq version.0.12.4[25] was used to quantify read coverage per gene. For all human CAFs RNA seq data we also performed the alignment pipeline (Trimmomatic – STAR – HTSeq) with hg38 reference genome. Next, Batch-effect corrections were performed by ComBat-seq[39].

GSVA analysis of three types of fibroblasts

Gene set enrichment was implemented using the R package 'GSVA' (function gsva - arguments: method = "gsva")[27]. By using a non-parametric unsupervised way of gene set enrichment, the relative enrichment of cell signatures of interest across the samples is computed. The result is GSVA enrichment scores of gene-set by sample matrix which are approximately normally distributed. GSVA generates enrichment score for each of gene sets such as GO EXTRACELLULAR MATRIX COMPONENT, GO EXTRACELLULAR MATRIX", GO EXTRACELLULAR MATRIX ASSEMBLY, GO EXTRACELLULAR STRUCTURE ORGANIZATION, REACTOME EXTRACELLULAR MATRIX ORGANIZATION, GO EXTRACELLULAR MATRIX STRUCTURAL CONSTITUENT CONFERRING TENSILE STRENGTH, GO CELL GROWTH, GO GROWTH FACTOR BINDING, HALLMARK ANGIOGENESIS, GO WOUND HEALING in the Molecular Signatures Database (MSigDB) with default parameters.

GSEA analysis of nine human CAFs with gene set of 'Cluster A' core regulatory circuit

Gene set enrichment analysis (GSEA) was performed using fgsea (fast GSEA) R-package with default parameters. For the enrichment analysis, we used 'Cluster A' gene set which is 174 genes whose enhancer contain motif of 10 TFs of 'Cluster A' core regulatory circuit (CRC). ('Cluster A' gene set : ADTRP, AHR, AKAP6, ARAF, ARID1A, ARID5B, ARNTL, BACH1, BCAS3, BHLHE40-AS1, BNC2, BTF3, C11orf68, CCDC85B, CLIC5, CREB3L1, CREB3L2, CUEDC1, DGKI, DLX2, DOK1, E2F7, EBF2, EBF3, EFCAB1, ETS1, FLJ22447, FOSL1, FOSL2, FOXC2, FOXL1, FOXO3, FOXP1, GAP43, GATA6, GATA6-AS1, GDF15, GLIS3, GLIS3-AS1, HIC1, HIF1A, HIF1A-AS2, HIVEP2, HLX, HM13-AS1, HNRNPA3, HOOK2, HOTAIRM1, HOXA2, HOXA3, HOXA4, HOXA-AS3, HOXB1, HOXB2, HOXB3, HOXB-AS1, HOXC4, HOXC6, HOXC8, ID2, ID3, JUN, JUNB, KCNJ15, KLF10, KLF4, KLF6, KLF7, KLF9, LINC00111, LINC00330, LINC00673, LINC00862, LINC00954, LINC01121, LINC01135, LINC01312, LINC01829, LINC01940, LOC100130880, LOC100505716, LOC100505795, LSM4, M1AP, MAPKAP1, MAX, MDS2, MEF2A, MEF2D, MEIS1, MEIS2, MIR1204, MIR2355, MIR3128, MIR3188, MIR3194, MIR4529, MIR4708, MIR5197, MIR548AV, MLXIP, MNT, MRPS23, MSL1, NAV2, NAV3, NFE2L1, NFIB, NKX2-6, NPAS2, NPAS3, NR1D1, NR2F2, NR3C1,

NR3C2, OACYLP, OSR1, PCAT1, PIGV, POU5F1B, PRDM1, PRDM8, PRKCH, PRRX1, PVT1, RARA, RARB, RERE, RNU6-16P, RREB1, RUNX1, RUNX2, SENCR, SGSM2, SKAP1, SMAD3, SMAD6, SMG6, SNAI2, SNAPC1, SNX13, SOX4, SOX9, SPIDR, STAT1, STAT4, STC1, SUMO1P1, TBX18, TCF12, TCF4, TCF7L2, TEAD1, TEX41, THRA, TMEM174, TNXB, TOM1L2, TOX2, TRPS1, VXN, ZBTB20, ZBTB38, ZBTB7A, ZEB2, ZEB2-AS1, ZFHX3, ZFHX4, ZFHX4-AS1, ZHX2, ZNF217, ZNF281, ZNF41, ZNF469)

Optimal cutoff of PRRX1 expression value for overall survival analysis

We determine the optimal cutpoint for prrx1 expression value using the maximally selected rank statistics from the 'maxstat' R package[40]. This was chosen a value of a cutpoint that correspond to the most significant relation with survival for possible cut-off points. The possible cut-off points was used in the range of 20th pecrentile to 80 percentil of prrx1 expression value.

Supplementary Fig. 1

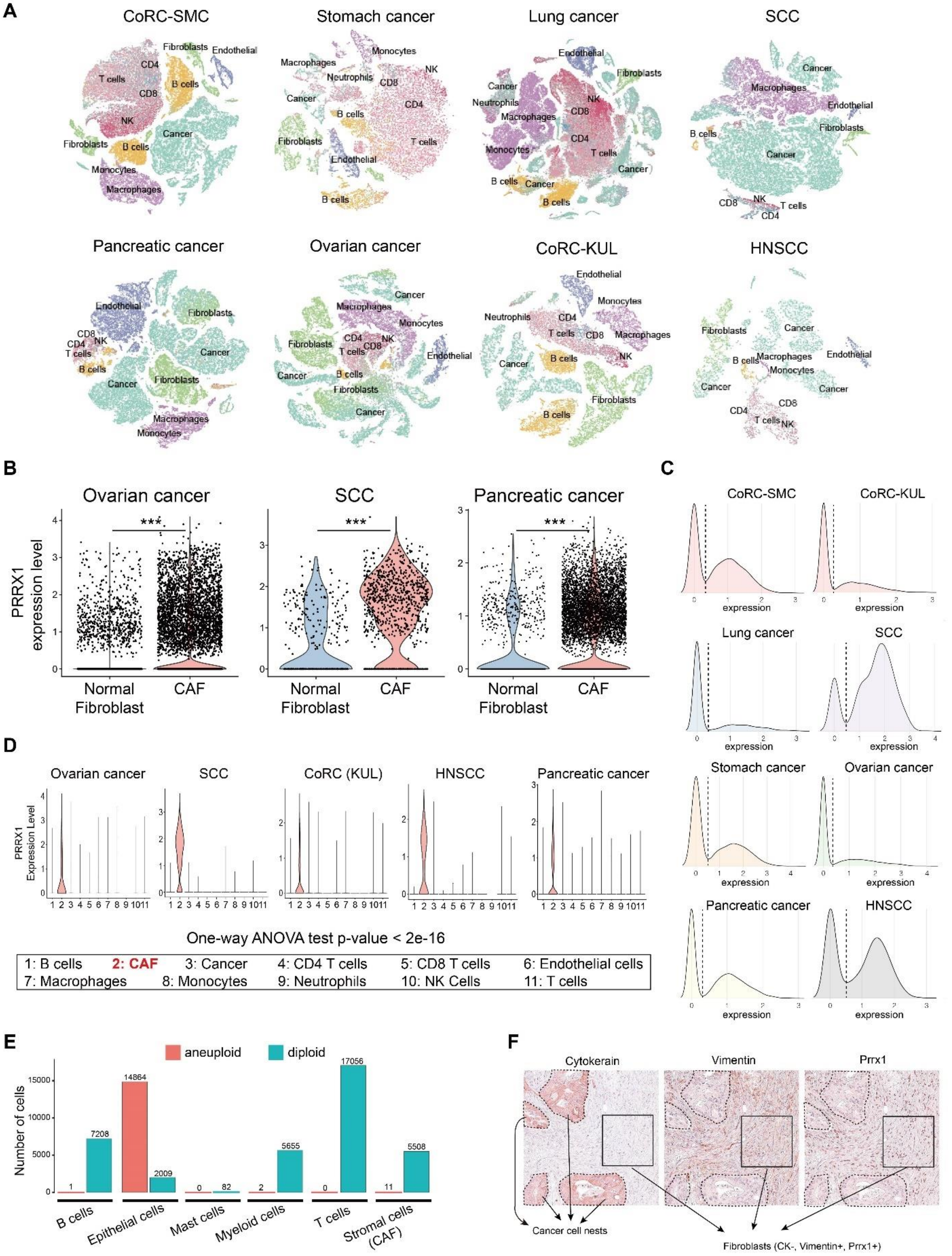

Supplementary Figure 1. *t*-SNE plots and correlation plots of single-cell RNA-seq obtained from total 8 dataset from 7 organs. (A) t-SNE plots colored by cell types designated by Cell Assign. We annotated individual cells for 11 cell types (B cells, fibroblasts, cancer cells, CD4 T cells, CD8 T cells, endothelial cells, macrophages, monocytes, neutrophils, NK cells, and T cells) in each of the eight datasets [colorectal cancer-SMC (CoRC-SMC), Stomach cancer, Lung cancer, Skin squamous cell carcinoma (SCC), Pancreatic cancer, Ovarian cancer, colorectal cancer -KUL (CoRC-KUL), Head & neck squamous cell carcinoma (HNSCC)]. **(B)** The level of *PRRX1* expression is illustrated in normal fibroblasts and cancer-associated fibroblasts (CAFs) using scRNA-seq datasets of ovary (n=8891), skin (n=812), and pancreas (n=8513) (p value was calculated using two-sided Wilcoxon rank-sum test: *** p < 2e-16). **(C)** Ridge plots show the criteria for dividing CAFs in *PRRX1* high (+) and low (-). **(D)** Analysis of scRNA-seq dataset revealed that *PRRX1* expression is highly restricted to CAFs among 11 cell types of ovarian, skin, pancreatic, colorectal, and head & neck squamous cell carcinoma (one-way ANOVA, p < 2e-16). **(E)** Single cells from the scRNA-seq data were classified into aneuploid or diploid cells using CopyKAT algorithm. From these results, most of the aneuploid cells were cancer cells. Among the CAF cells, only 11 out of 5508 cells were aneuploid, indicating that CAFs derived from cancer cells were extremely rare. **(F)** Pan-cytokeratin, vimentin, and Prrx1 immunohistochemistry showed that Prrx1+/vimentin+ fibroblast with spindle shape in tumor stroma was not associated with cytokeratin + epithelial cancer cells. Images are representative of three tissue samples. Source data are provided as a Source Data file.

Supplementary Fig. 2

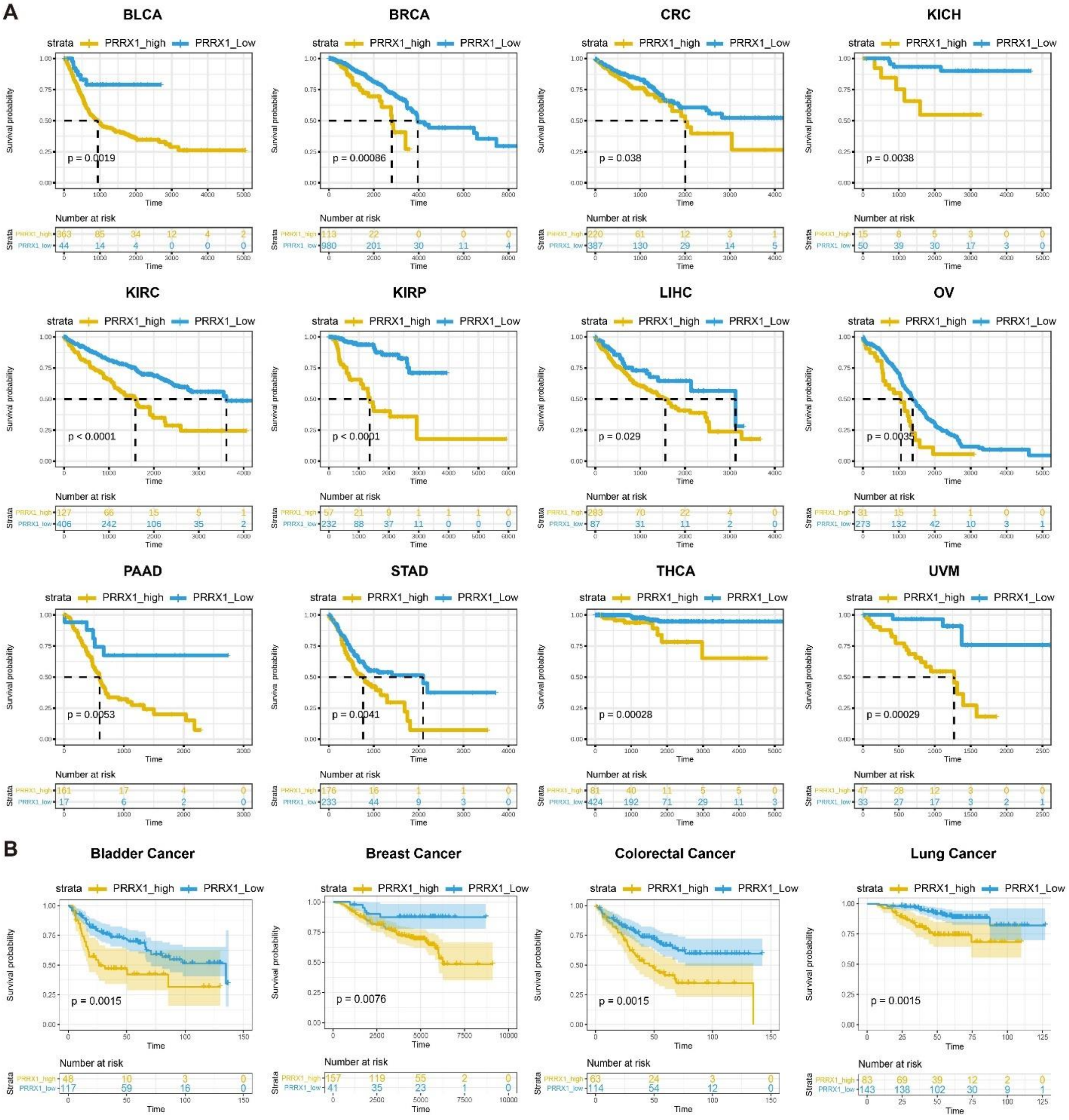

Supplementary Figure 2. Survival analysis identifies *PRRX1* expression level as a potential prognostic marker in multiple cancers (related to Figure 1F). (A) Survival analysis in TCGA patients. We clustered the patients into high- and low-PRRX1 groups and performed a Kaplan-Meier plot with log-rank test (see Supplementary Methods for the more details). The survival of the high-PRRX1 group was significantly lower than that of the low-PRRX1 group in 12 cancer types. **(B)** Survival analysis in bladder (GSE13507), breast (GSE7390), colorectal (GSE17536),

and lung (GSE31210) cancer patients. We analyzed the same as in (A) and also found that the survival of the high-PRRX1 group was significantly lower than that of the low-PRRX1 group in the four datasets. Survival curves with 95% confidence interval are provided.

Supplementary Fig. 3

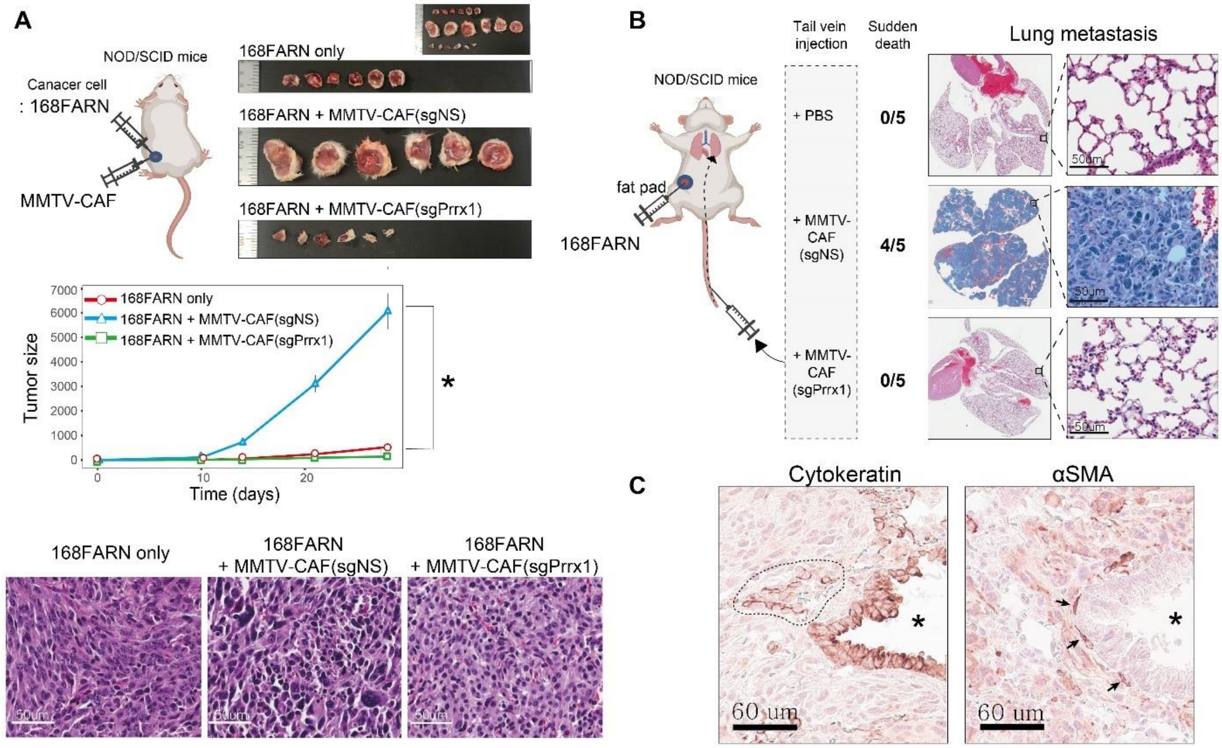

Supplementary Figure 3. Prrx1 enhances tumor progression and metastasis of various tumor cells *in vivo* through the activation of co-implanted CAFs in immunodeficient mice. **(A)** Mouse breast cancer cells (168FARN) alone or mixed with WT MMTV-CAFs (sgNS) or Prrx1-deficient MMTV-CAFs (sgPrrx1) were co-injected subcutaneously into NOD-SCID mice (n = 6 mice in each group). Error bars, SEM; *p* value was calculated using two tailed t-test: $*p <$ 0.0001; Scale bar, 50 µm. **(B)** Cancer cells (168FARN) were injected into the mammary fat pad of NOD-SCID mice. MMTV-CAFs (sgNS or sgPrrx1) were intravenously injected into the mice (n = 5 mice in each group). PBS was used as a control. Scale bar, 50 µm. **(C)** CK (cytokeratin) and αSMA staining images of a representative lung metastasis. CK and αSMA were not stained in the lungs of mice injected with CAF sgprrx1 into the tail vein (no lung metastases), whereas CK (as an indicator of 168FARN) and αSMA (as an indicator of MMTVCAF) were observed in the lungs of mice injected with CAF sgNS into the tail vein. Images are representative of three tissue samples. Source data and exact p values are provided as a Source Data file.

Supplementary Fig. 4

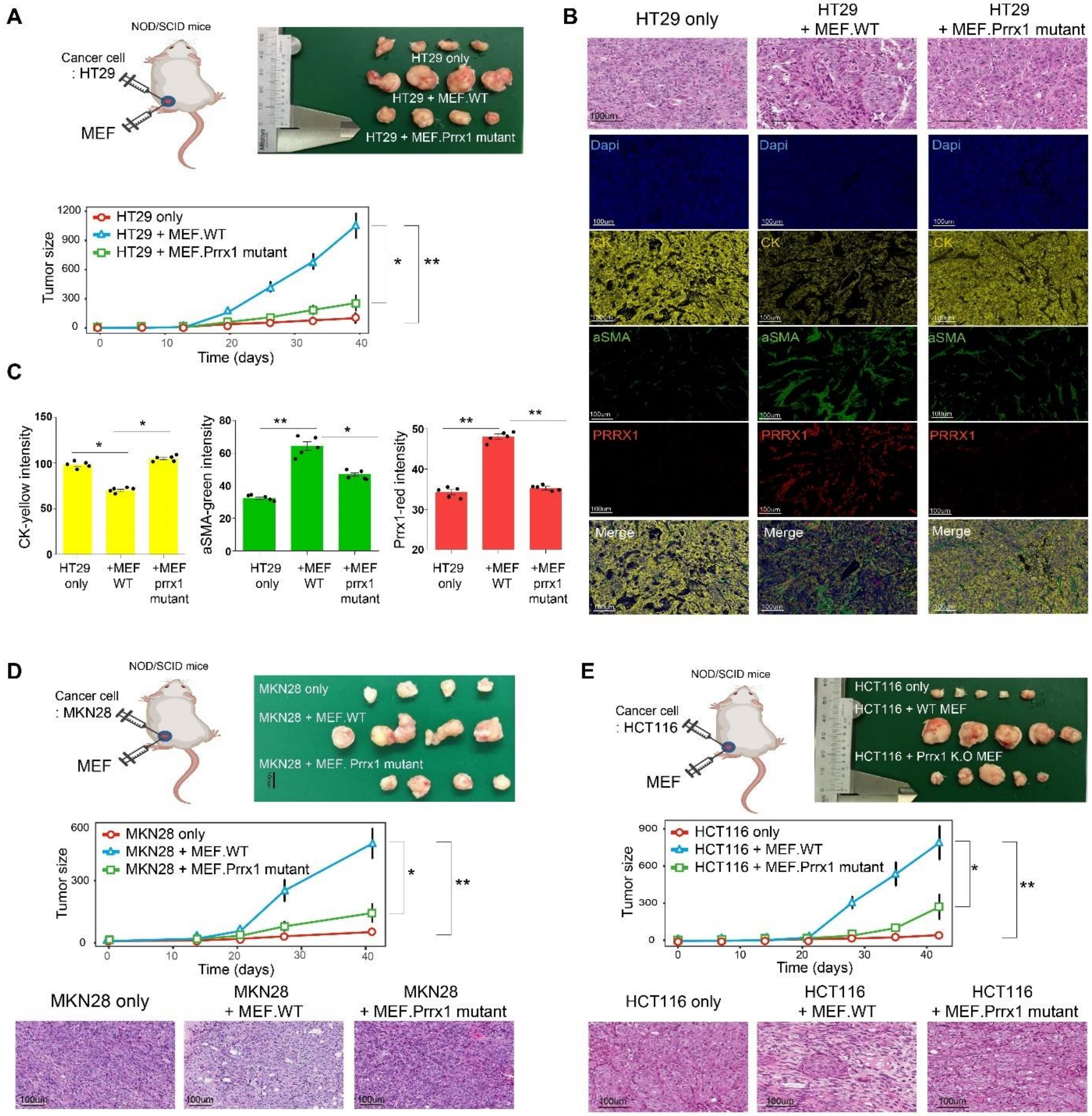

Supplementary Figure 4. Prrx1 status in fibroblasts alters tumor progression in various co-graft mice models. (A) HT29 human colon cancer cells alone or mixed with WT or Prrx1 mutant primary MEFs were co-injected subcutaneously into NOD/SCID mice (n = 4 mice in each group) and tumor volume was measured. Error bars, SEM; *p* value was calculated using two tailed t-test: **p* < 0.005; ***p* < 0.001. **(B, C)** H&E and IHC staining of a representative primary tumor. IHC staining was visualized and quantified using ImageJ Fiji [DAPI (heamatoxylin)-blue, αSMA-green, cytokeratin-yellow, Prrx1-red]. Scale bar, 100 μm. Each intensity of IHC representing the

expression level of each gene was measured using ImageJ Fiji. Data are presented as the mean ± SEM; N = 5 independent measurements (two-tailed t test: *p<0.0001, **p<0.0005). **(D)** MKN28 human stomach cancer cells alone or mixed with WT or Prrx1 mutant primary MEFs were co-injected subcutaneously into NOD/SCID mice (n = 4 mice in each group) and tumor volume was measured. H&E images in each group are shown. Error bars, SEM; *p* value was calculated using two tailed t-test: $*p < 0.01$; $**p < 0.005$. **(E)** HCT116 human colon cancer cells alone or mixed with WT or Prrx1 mutant primary MEFs were co-injected subcutaneously into NOD/SCID mice (n = 5 mice in each group) and tumor volume was measured. H&E images in each group are shown. Error bars, SEM; *p* value was calculated using two tailed t-test: $*p < 0.05$; $**p < 0.001$. Source data and exact p values are provided as a Source Data file.

Supplementary Fig. 5

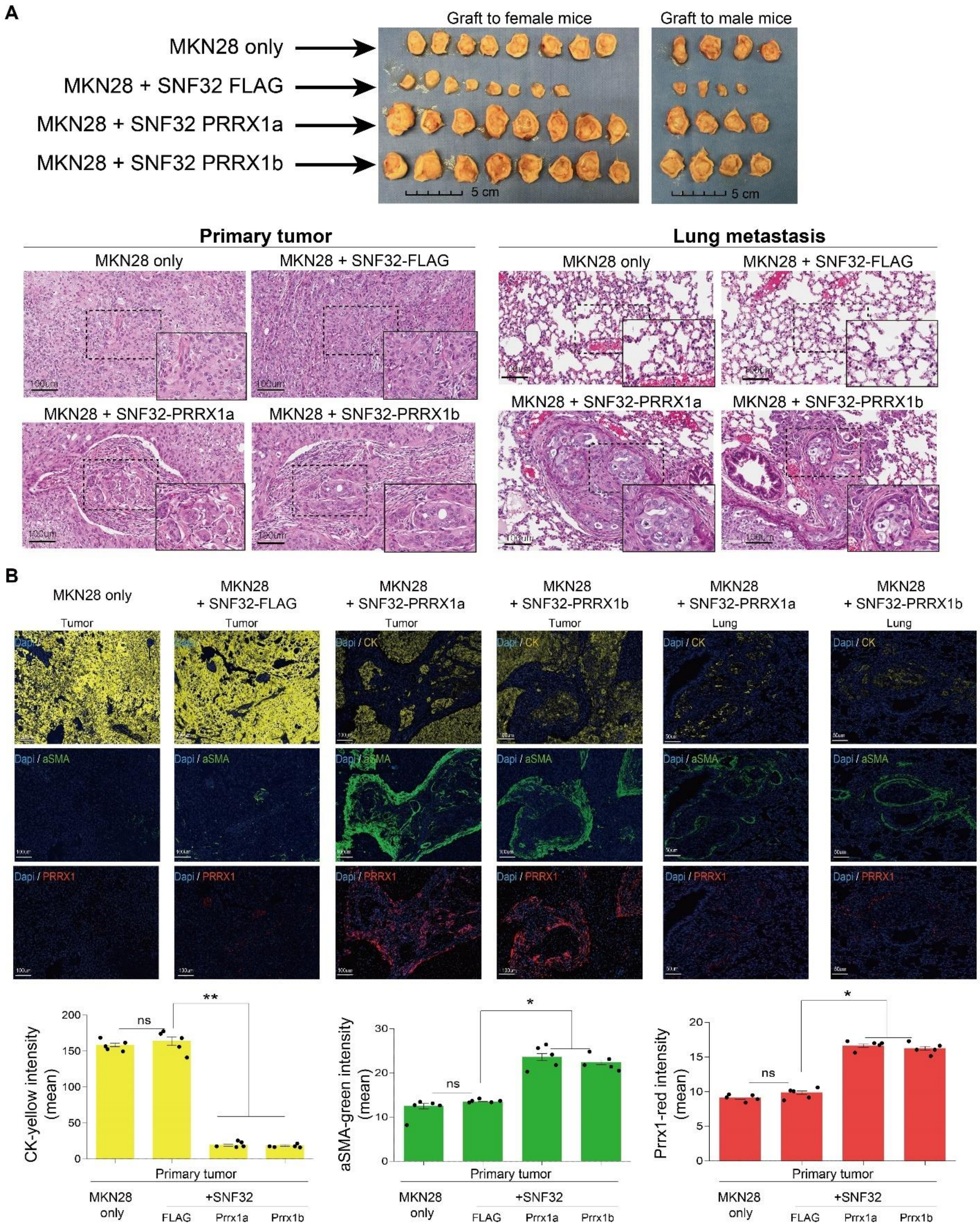

Supplementary Figure 5. Prrx1 status in fibroblasts alters tumor progression in various co-graft mice models. (A) MKN28 human stomach cancer cells alone or mixed with SNF32 (human stomach normal fibroblasts)-FLAG (control) or SNF32 overexpressing Prrx1a or Prrx1b were co-injected subcutaneously into both flanks of six NSG mice. n = 8 tumors per group in female mice, n=4 tumors per group in male mice (see Figure 1G-1I). Representative H&E images of primary tumor and lung metastasis are shown. **(B)** Immunohistochemical (IHC) staining of a representative primary tumor and lung metastasis. Visualization of IHC staining using ImageJ Fiji [DAPI (heamatoxylin)-blue, αSMA-green, cytokeratin-yellow, Prrx1-red] are shown. Primary tumor images: scale bar, 100 µm. Lung metastasis images: scale bar, 50 µm. Each intensity of IHC representing the expression level of each gene was measured using ImageJ Fiji. Data are presented as the mean ± SEM; n = 5 independent measurements (p value was calculated using two tailed t-test: *$p < 0.05$; **$p < 0.001$). Source data and exact p values are provided as a Source Data file.

Supplementary Fig. 6

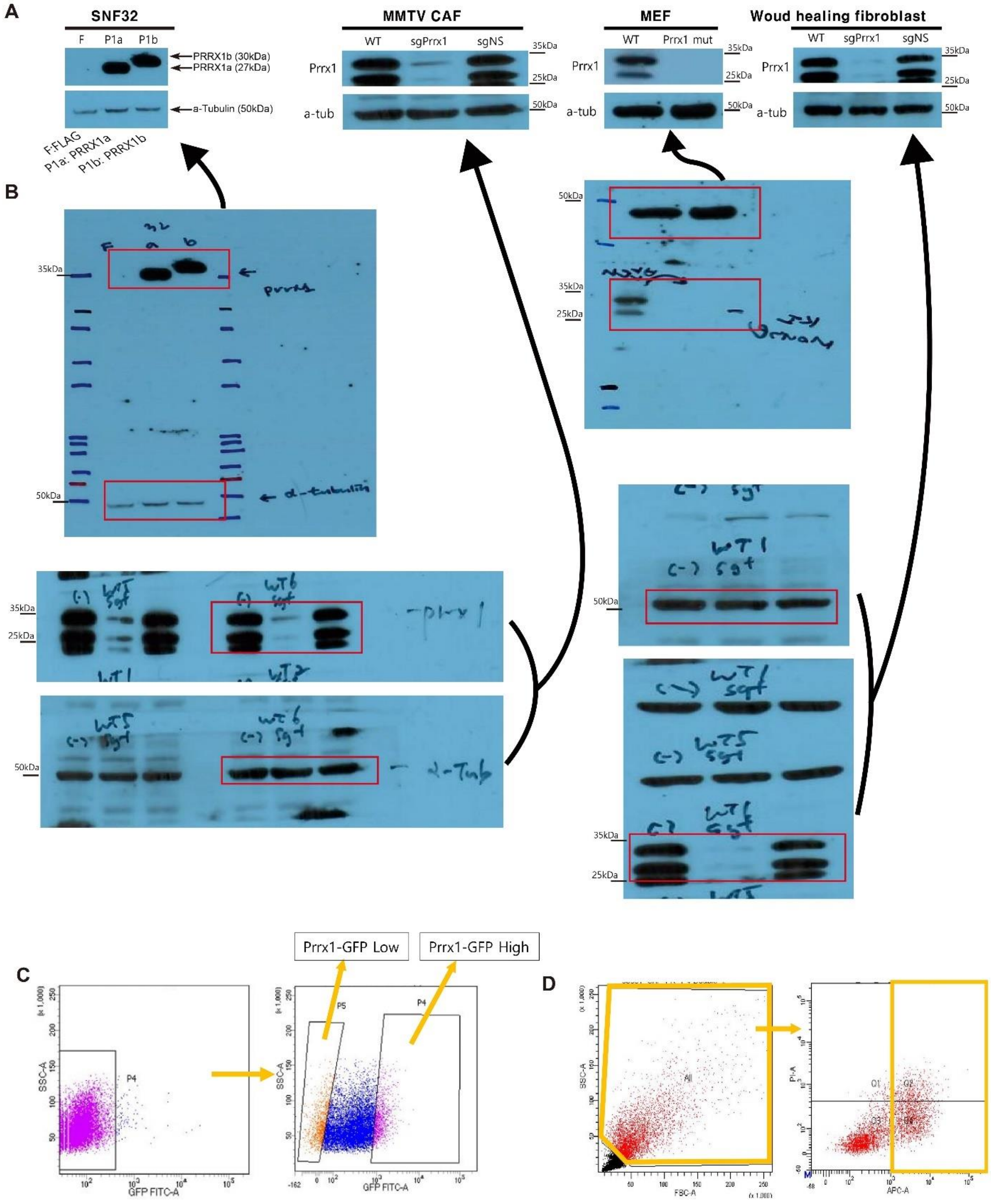

Supplementary Figure 6. Uncropped images of western blots corresponding to the indicated blots and FACS sorting/gating strategy. (A) Western blot analysis of endogenous Prrx1 isoforms and exogenous Prrx1 expression. Whole cell lysates were subjected to western blotting for Prrx1 and a-Tubulin (related to figure 1G and figure 3A). **(B)** Uncropped Western blot images are shown that correspond to Supplementary Figure 6A. Cropped images are marked with a red box. **(C)** CAF cells were infected lentivirus containing prrx1 promoter-GFP reporter. FACS sorting was performed to isolate FITC population(GFP high and GFP low) in experiments including figure 3B. **(D)** Annexin V+ / Propidium Iodide- or + cell analysis by FACS: Analysis strategy shown above was used to determine the apoptotic cell population in all experiments.

Supplementary Fig. 7

A

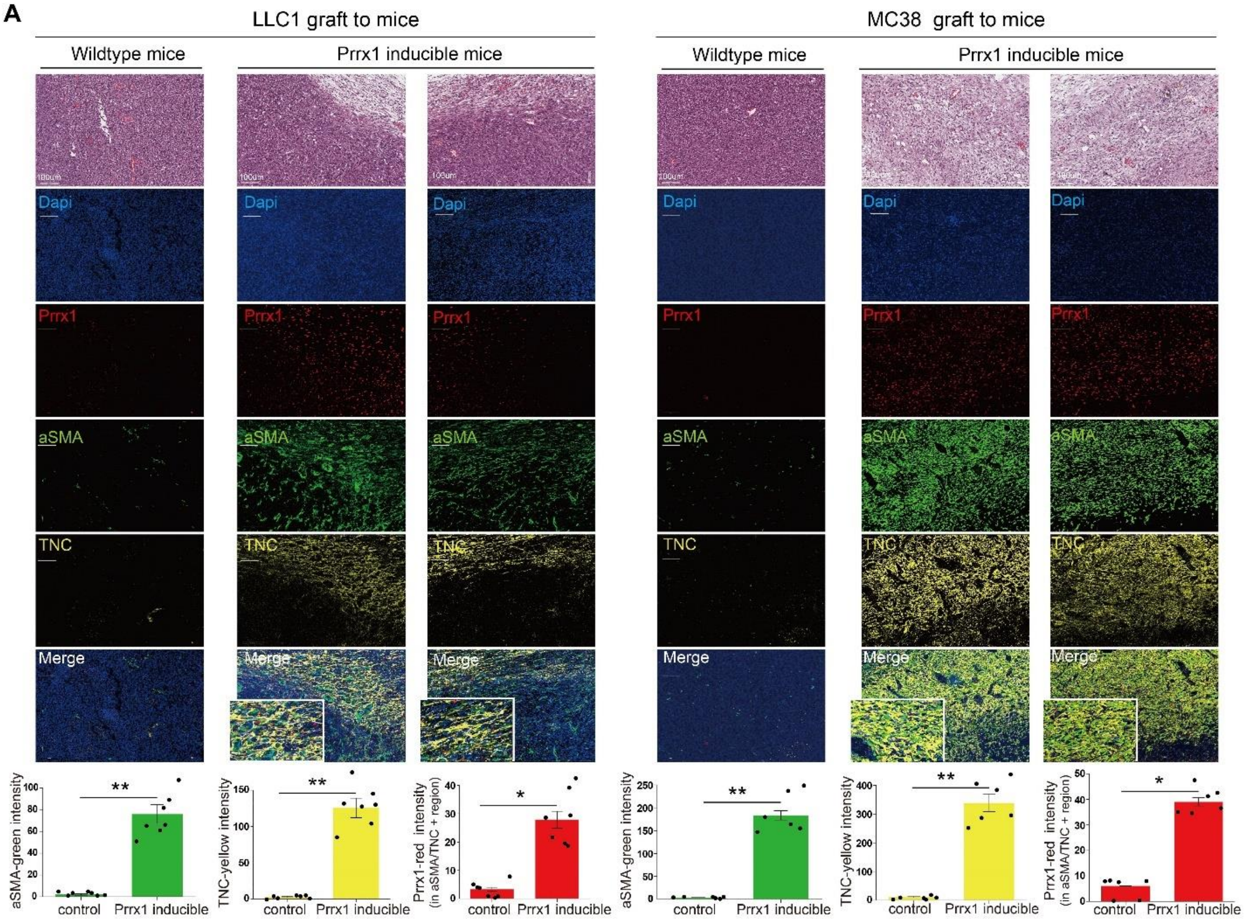

B

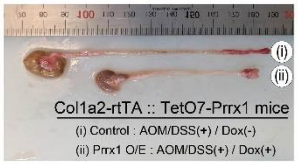

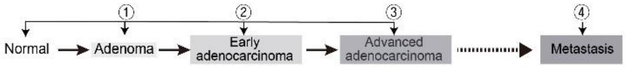

O/E: Over-expression

	Number of mice	Adenoma	Early adenocarcinoma	Advanced adenocarcinoma	Metastasis
Control : AOM/DSS(+), Dox(-)	8	87.5% (7/8)	12.5% (1/8)	0% (0/8)	0% (0/8)
Prrx1 O/E : AOM/DSS(+), Dox(+)	13	23.08% (3/13)	61.54% (8/13)	15.38% (2/13)	15.38% (2/13)

C

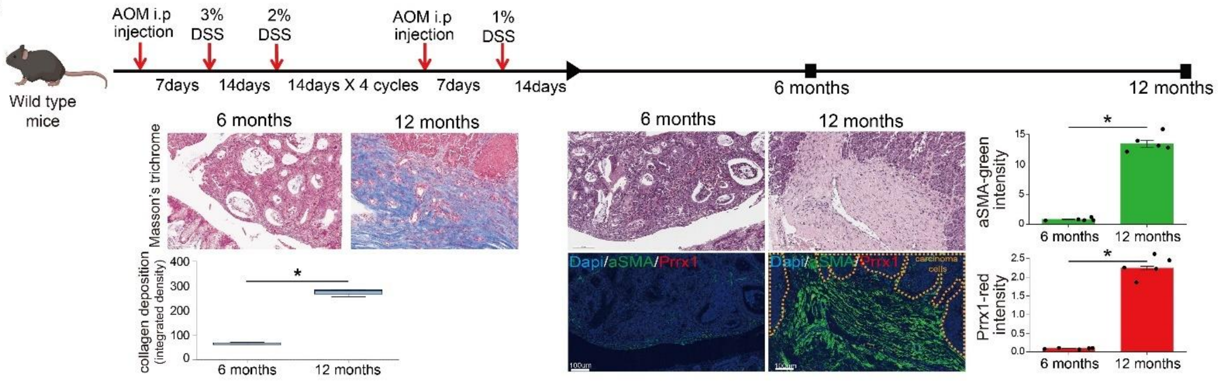

Supplementary Figure 7. Prrx1 in fibroblasts is indispensable for tumor formation *in vivo* and is sufficient to promote metastasis. (A) LLC1 tumors from WT and fibroblast-specific Prrx1-induced mice (Col1a2rtTA;TetO7-Prrx1Luc) (see Figure 2B). H&E and IHC staining images of representative LLC1 Luc-GFP primary tumor in each group. IHC staining images were visualized using ImageJ Fiji [DAPI (heamatoxylin)-blue, αSMA-green, TNC-yellow, Prrx1-red]. Scale bar, 100 µm. In Col1a2rtTA;TetO7-Prrx1Luc mice, Prrx1 was induced in fibroblasts after skin transplantation of mouse colon cancer cells MC38-Luc-GFP. In these mice, skin excision and tumor removal surgery were performed after tumor formation. Primary tumors in WT (-Dox) control and Prrx1-inducible (+Dox) mice (see Figure 2C). H&E and IHC staining images of representative MC38-Luc-GFP primary tumor in each group. IHC images were visualized using ImageJ Fiji [DAPI (heamatoxylin)-blue, αSMA-green, TNC-yellow, Prrx1-red]. Scale bar, 100 µm. Each intensity of IHC representing the expression level of each gene was measured using ImageJ Fiji. Data are presented as the mean ± SEM; n = 6 independent measurements (*p* value was calculated using two tailed t-test: *p<0.0001, **p<0.0005). **(B)** Timeline for *in vivo* model of AOM/DSS-induced colorectal cancer (see Figure 2D). Comparison of the incidence rate of each stage ranging from adenoma to metastasis induced by AOM/DSS treatment in WT and fibroblast-specific Prrx1-inducible (Col1a2^{rtTA}; TetO7-Prrx1^{Luc}) mice. **(C)** Timeline for AOM/DSS-induced colorectal cancer *in vivo* model. One group was treated with AOM/DSS for 6 months to observe adenoma and early adenocarcinoma (early stage), while the other group was treated with AOM/DSS for 1 year to observe advanced adenocarcinoma (advanced stage). H&E, Masson's trichrome, and IHC staining images of representative colon tissue in each group. IHC staining images were visualized using ImageJ Fiji [DAPI (heamatoxylin)-blue, αSMA-green, Prrx1-red] (n = 10 mice per group). Scale bar, 100 µm. Each intensity of IHC representing the expression level of each gene was measured using ImageJ Fiji. Data are presented as the mean ± SEM; n =5 independent measurements (*p* value was calculated using two tailed t-test: *p<0.0001). Source data and exact p values are provided as a Source Data file.

Supplementary Fig. 8

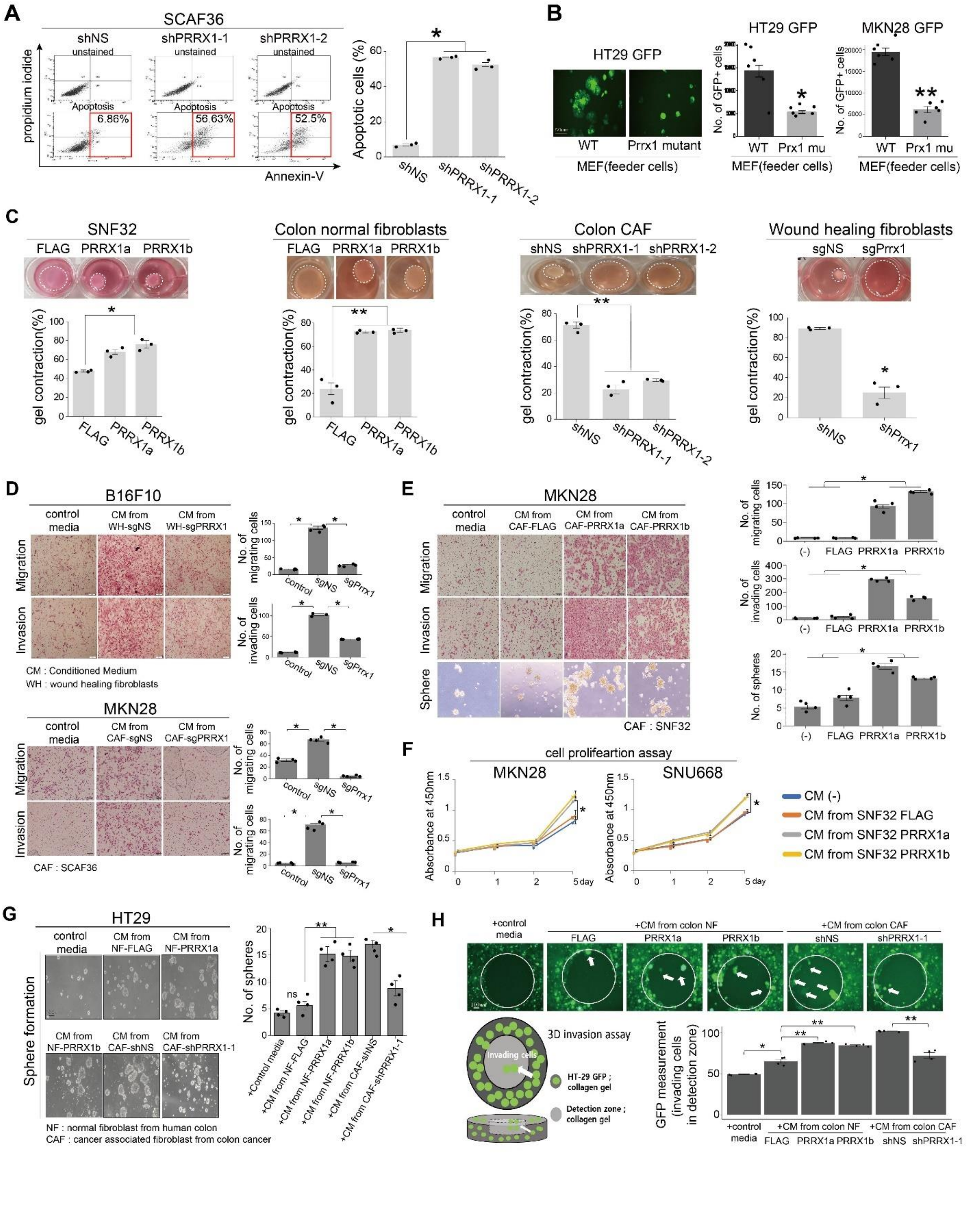

Supplementary Figure 8. Prrx1 orchestrates various functions of CAFs that enhance tumor EMT and stemness. (A) Apoptosis assay on human stomach CAF (SCAF36) after knockdown of *PRRX1*. The rate of cellular apoptosis increased significantly after knockdown of *PRRX1*. The FACS-based apoptosis assay was repeated in triplicate. Data are presented as the mean ± SEM; n =3 independent measurements (*p* value was calculated using two tailed t-test: *p<0.0001). **(B)** WT or Prrx1 mutant MEFs (mouse embryonic fibroblasts) were treated with 10 mg/mL mitomycin C. HT29 human colon cancer cells and MKN28 human stomach cancer cells stably expressing GFP were seeded on top of MEFs (feeder cells) and allowed to grow for 3–4 days, after which cancer cells were trypsinized and quantificated using flow cytometry. Representative image of GFP^+ HT29 cell growth on top of MEFs. The mean of independent measurements(n=6) is shown. Error bars, SEM; *p* value was calculated using two tailed t-test: *$p < 0.05$, **$p < 0.001$. **(C)** Contraction assay: *PRRX1* overexpression in various normal fibroblasts improved contraction ability. Knockdown of Prrx1 in various activated fibroblasts (mouse wound healing fibroblasts and human colon CAFs) reduced contraction ability. The gel contraction assay was repeated in triplicate. Data are presented as the mean ± SEM; n=3 independent experiments (*p* value was calculated using two tailed t-test: *p<0.01, **p<0.001). **(D, E)** B16F10 mouse melanoma cells were cultured alone (-) or with CM of mouse wound healing fibroblasts SgNS or SgPrrx1 (Prrx1 deleted). The same experiments with MKN28 and SCAF36 (human stomach CAFs) ShNS or ShPRRX1. The abilities of proliferation, migration invasion, sphere formation of cancer cells (MKN28, SNU668) were significantly enhanced after incubation with CM derived from $PRRX1^+$ fibroblasts (mouse wound healing fibroblast SgNS and SCAF36 ShNS), but these increased abilities disappeared when cultured with CM from Prrx1-deficient fibroblasts (mouse wound healing fibroblasts SgPrrx1 and SCAF36 ShPRRX1). For D and E : Data are presented as the mean ± SEM; n =4 independent experiments (*p* value was calculated using two tailed t-test: *p<0.001). **(F)** In addition, these abilities of cancer cells (MKN28 and SNU668) were greatly increased when cultured with CM from SNF32 overexpressing Prrx1a or Prrx1b. This experiment was measured in four replicates. Error bars, SEM; *p* value was calculated using two tailed t-test: *p<0.0001. **(G, H)** The abilities of sphere formation (left,) and invasion (right,) of HT29 cancer cells were enhanced when cultured with CM from $PRRX1^+$ fibroblasts (colon NF overexpressing PRRX1a or PRRX1b, colon CAF ShNS). For G : Data are presented as the mean ± SEM; n =5 independent experiments. For H : Data are presented as the mean ± SEM; n =4 independent experiments. *p* value was calculated using two tailed t-test: *p<0.001, **p<0.0001. Source data and exact p values are provided as a Source Data file.

Supplementary Fig. 9

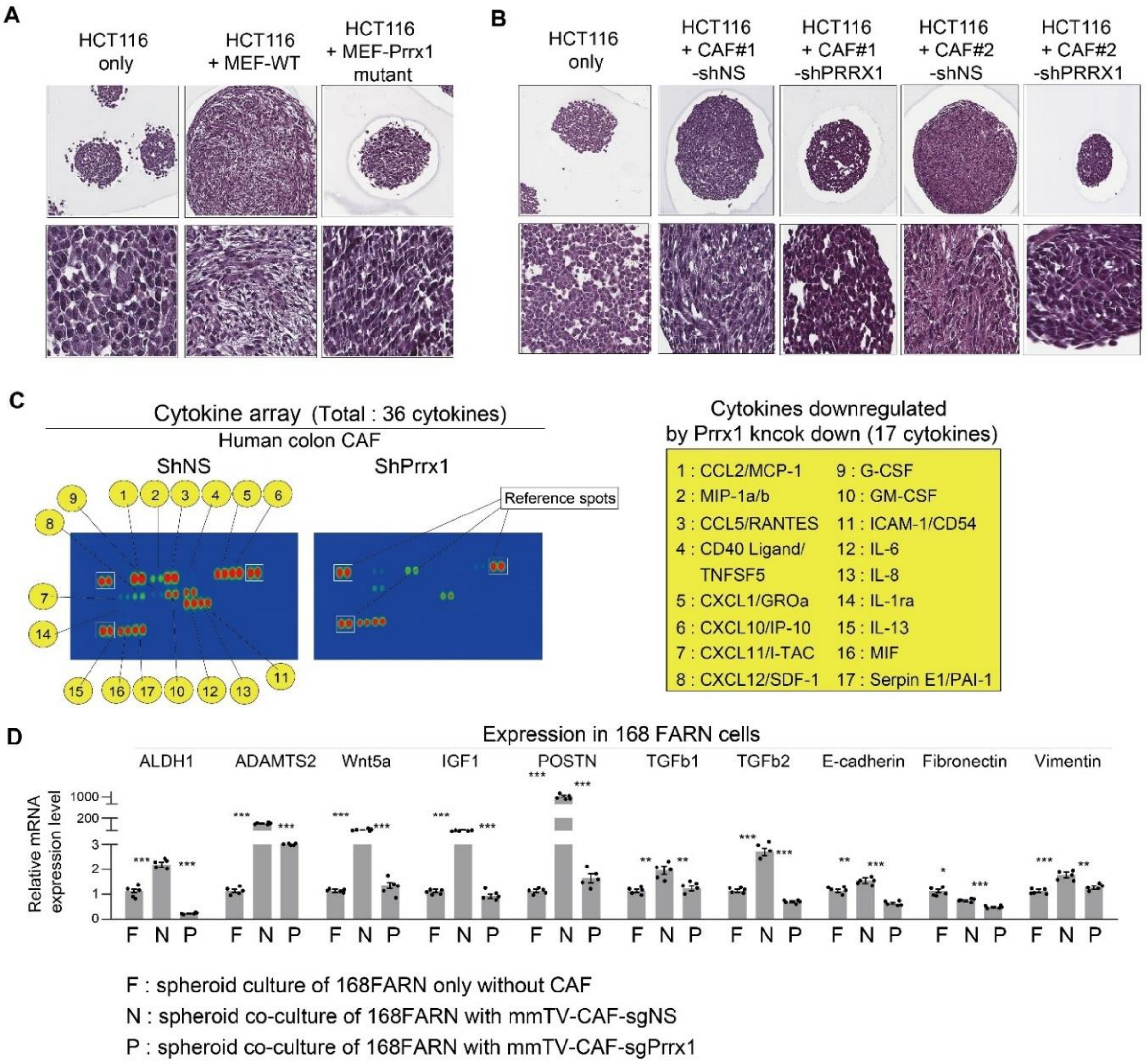

Supplementary Figure 9. Prrx1 orchestrates various functions of CAFs that enhance tumor EMT and stemness. (A-B) Hanging drop direct spheroids co-culture system. The spheroid size was increased in the group formed by cancer cells and PRRX1⁺ fibroblasts (WT MEFs and colon CAF ShNS) and cancer cells in this group showed a more spindle-shaped phenotype, as if EMT was induced compare to the other groups. This spheroid experiment was independently repeated three times with similar results. **(C)** Cytokine array was performed using conditioned media. After knockdown of *PRRX1* in colon CAFs, various secreted proteins that can affect cancer cells were significantly reduced. **(D)** The RNA-Seq results shown in Figure 3G were confirmed using qPCR for several genes representing changes in EMT, stemness, metastasis, and proliferation of cancer cells. Error bars, SEM; n =5 experiments, *p* value was calculated using two tailed t-test: *p < 0.05; **p < 0.005; ***p <0.001. Source data and exact p values are provided as a Source Data file.

Supplementary Fig. 10

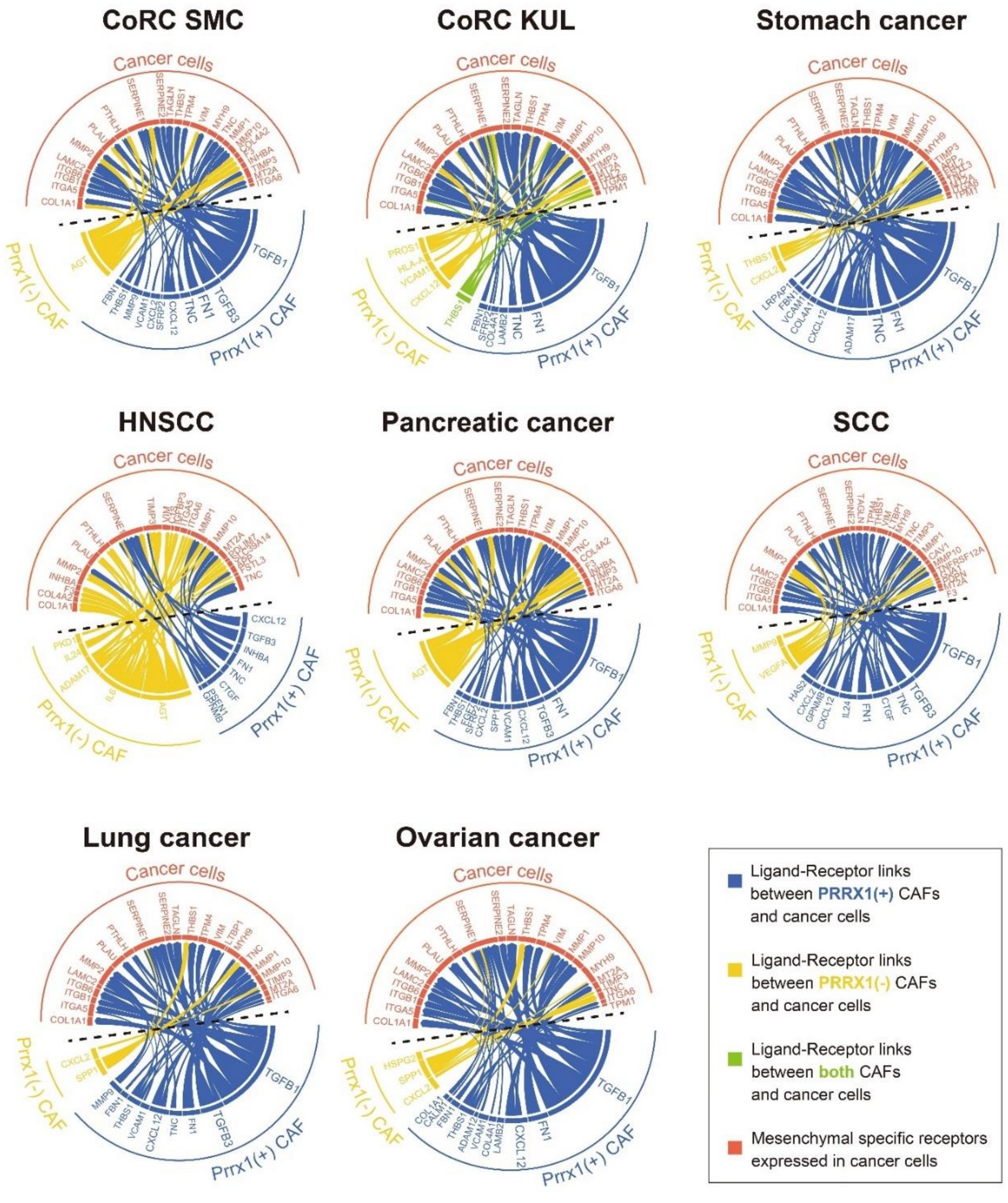

Supplementary Figure 10. Inference on interactions between EMT-like cancer cells and CAFs shows that high PRRX1-expressing CAFs interacted more with EMT-like cancer cells. Circus plots show inferred ligand-receptor links between EMT-associated receptors in cancer cells (red) and high PRRX1-expressing (blue) and low PRRX1-expressing (yellow) CAFs. Common links of both CAFs are shown in green. The bar plots show the number of links in the circus plots. Source data are provided as a Source Data file.

Supplementary Fig. 11

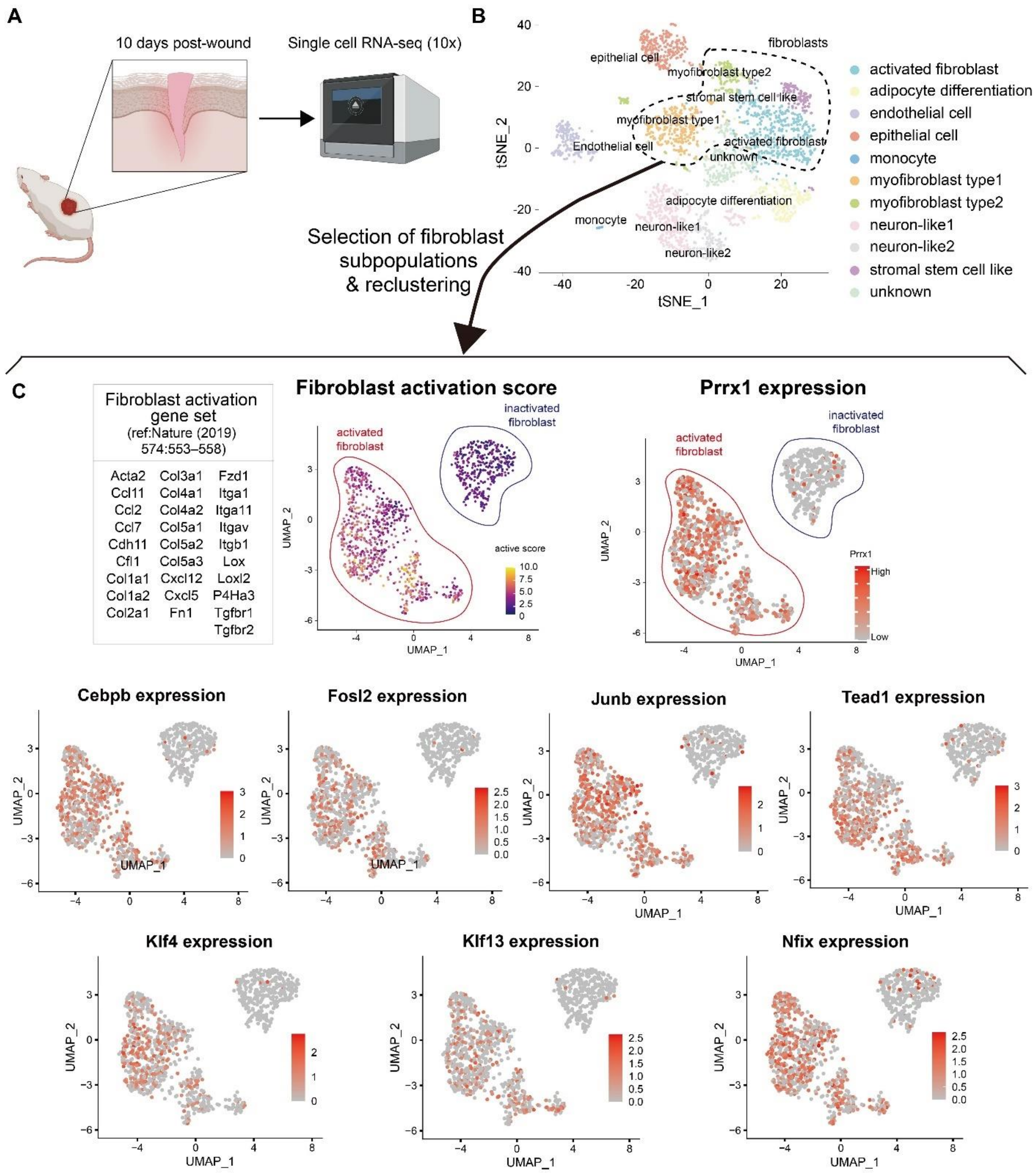

Supplementary Figure 11. Single-cell RNA-seq (scRNA-seq) from 10 days post-wound tissues revealed that the master TFs including Prrx1 were highly expressed in activated fibroblast. (A) Schematic diagram of scRNA-seq of 10 days post wound tissue from C57BL/6 mouse. **(B)** Uniform Manifold Approximation and Projection (UMAP) plot shows 11 cell types identified by clustering analysis with previously reported signatures. Then, fibroblasts clusters (myofibroblast types1, 2, stromal stem cell-like cell, and activated fibroblast) were isolated. **(C)** Using fibroblast activation gene set, we calculated fibroblast activation score. UMAP visualized the expression of fibroblast activation score and expressions of eight murine common CRC TFs. These TFs were highly expressed in the activated fibroblast cluster. CRC, core-regulatory circuitry; TF, transcription factor.

Supplementary Fig. 12

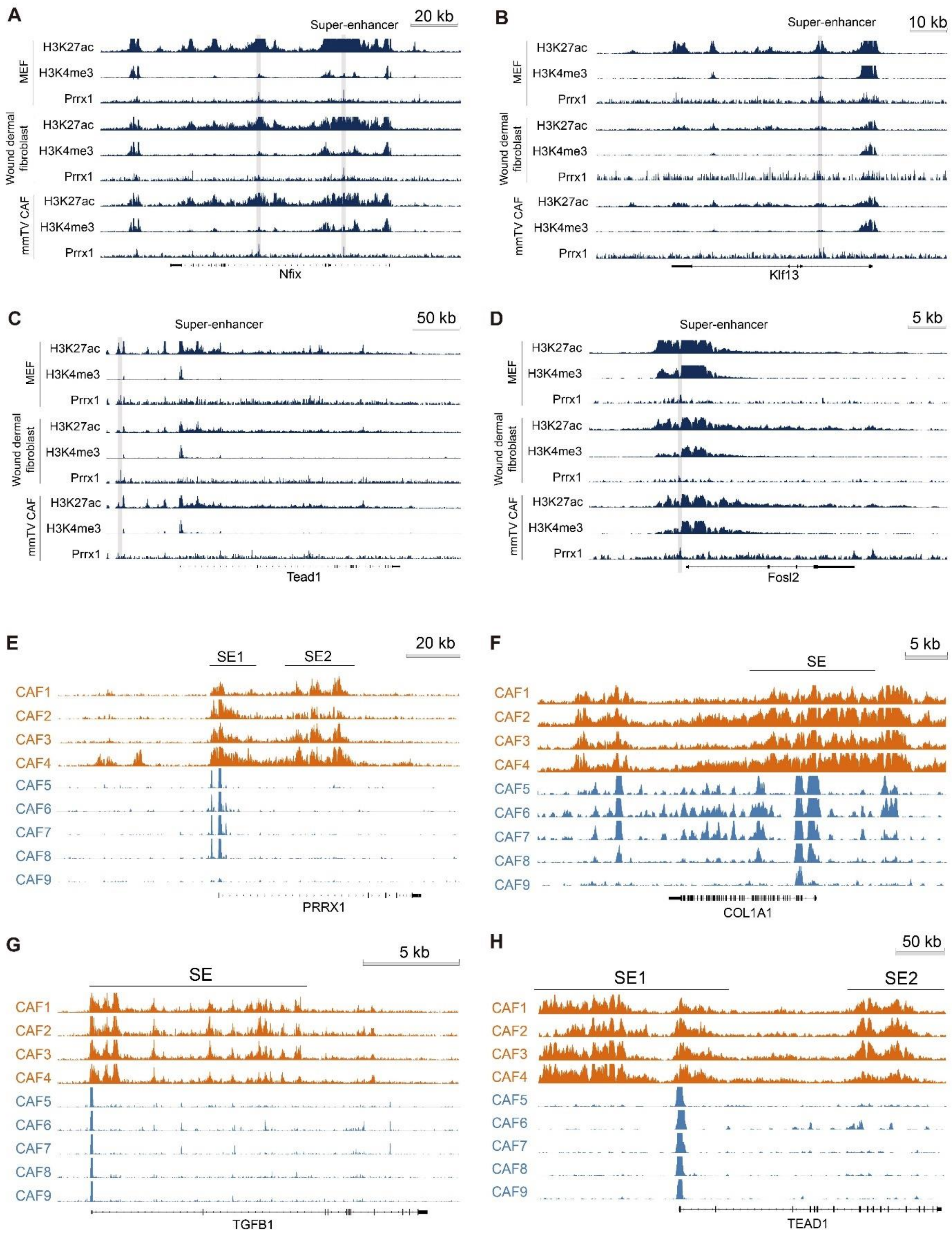

Supplementary Figure 12. H3K27ac ChIP-seq, H3K4me3 ChIP-seq, and Prrx1 ChIP-seq profiles of representative myofibroblast-specific genes for murine fibroblast and all nine CAFs. (A-D) H3K27ac ChIP-seq, H3K4me3 ChIP-seq, and Prrx1 ChIP-seq profiles of representative CRC TFs of activated murine fibroblasts. **(E-H)** H3K27ac ChIP-seq profiles of genes related to phenotypes of myofibroblastic CAFs such as *COL3A1* (major extracellular matrix component), *TGFB1*(TGF-β signaling), *TEAD1* (activated fibroblast and contractile) are shown for all nine CAFs. The super-enhancer regions are indicated at the top by bars. CRC, core-regulatory circuitry; TF, transcription factor.

Supplementary Fig. 13

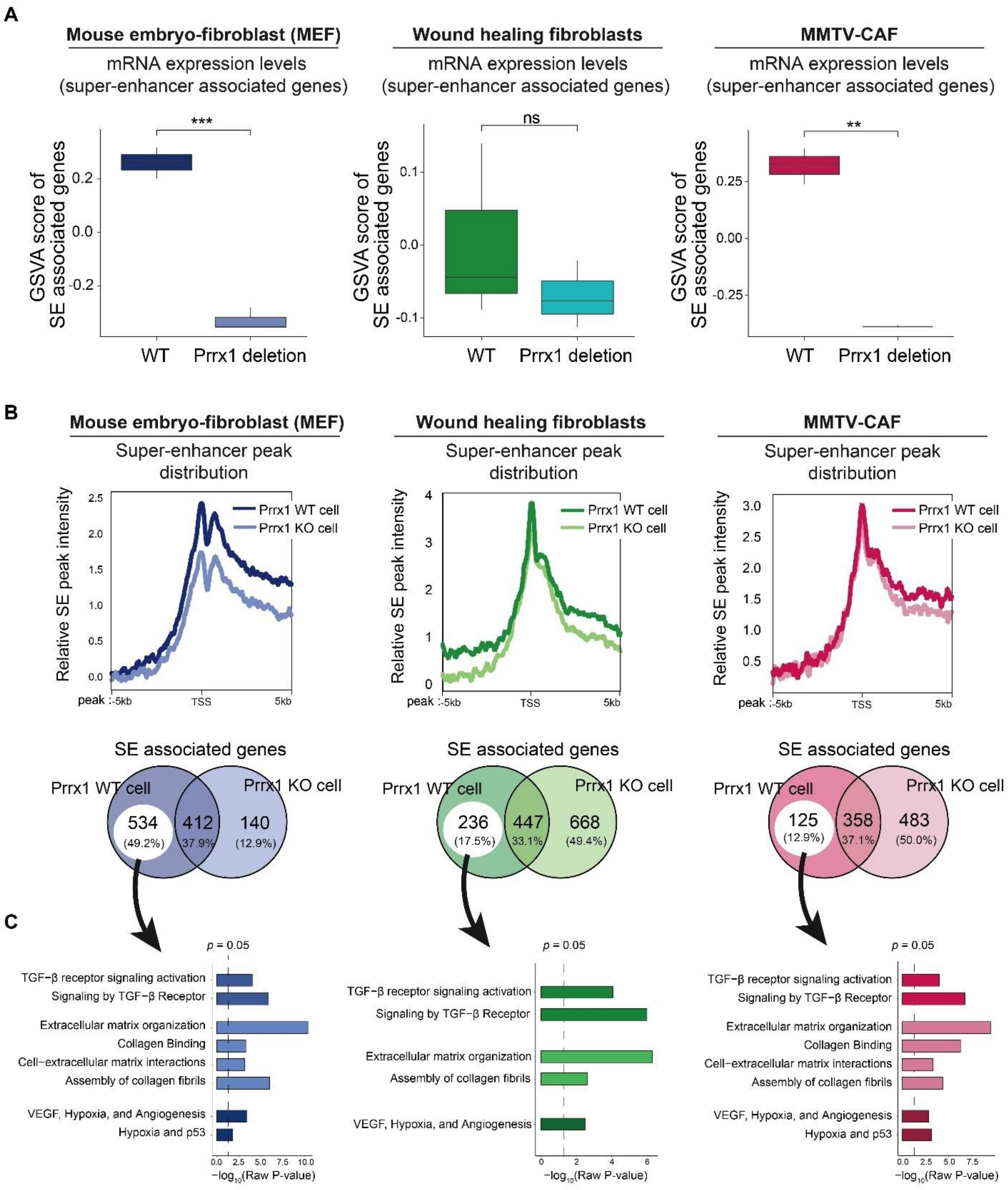

Supplementary Figure 13. Validations of super-enhancer using RNA-seq and illustration of super-enhancer of three types of WT Prrx1 or Prrx1 mutant fibroblasts. (A) Boxplots show that significant association of GSVA scores of super-enhancer (SE) associated genes according to WT Prrx1 and Prrx1 mutant fibroblasts. These SE associated genes are enriched in Prrx1 WT murine fibroblasts (*p* value was calculated using two-sided Wilcoxon rank-sum test: ns : not significant, ** p < 1e-2, *** p < 1e-3). **(B)** Super-enhancer peak distribution plots of three types of WT Prrx1 and mutant Prrx1 (top). Venn diagram shows the inclusion of SE-related genes in WT Prrx1 and Prrx1 mutant (bottom). **(C)** GO analysis show that SE associated genes specific to WT Prrx1 murine fibroblasts are enriched in activated fibroblast phenotypes such as Extracellular organization, TGF-β signaling, angiogenesis, and hypoxia. Source data and exact p values are provided as a Source Data file.

Supplementary Fig. 14

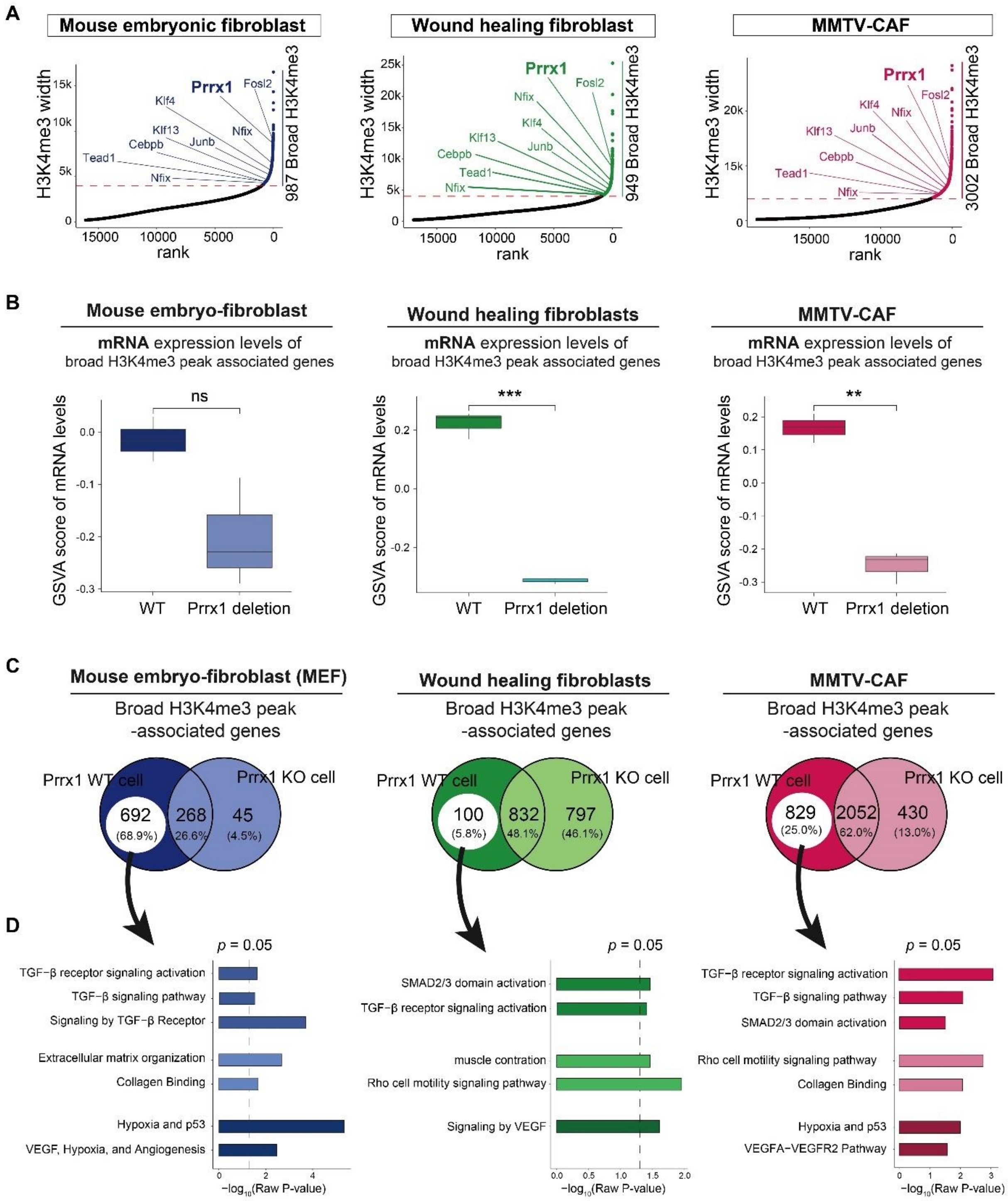

Supplementary Figure 14. Broad H3K4me3 peaks in three types of mouse fibroblasts and molecular functions associated with these peaks. (A) Distribution of H3K4me3 breadth in three types of fibroblasts, with a subset of exceptionally broad H3K4me3 peaks, which is more wider that 4 kb. **(B)** Significant association of GSVA scores for broad H3K4me3 specific genes according to WT Prrx1 and Prrx1 mutant fibroblasts. The Broad H3K4me3 associated genes are also enriched in WT Prrx1 murine fibroblasts (*p* value was calculated using two-sided Wilcoxon rank-sum test: ns : not significant, ** p < 1e-2, *** p < 1e-3). **(C, D)** GO analysis show that broad H3K4me3 associated genes specific of WT Prrx1 murine fibroblasts are enriched in activated fibroblast phenotypes such as Extracellular organization, TGF-β signaling, angiogenesis, and hypoxia. Source data and exact p values are provided as a Source Data file.

Supplementary Fig. 15

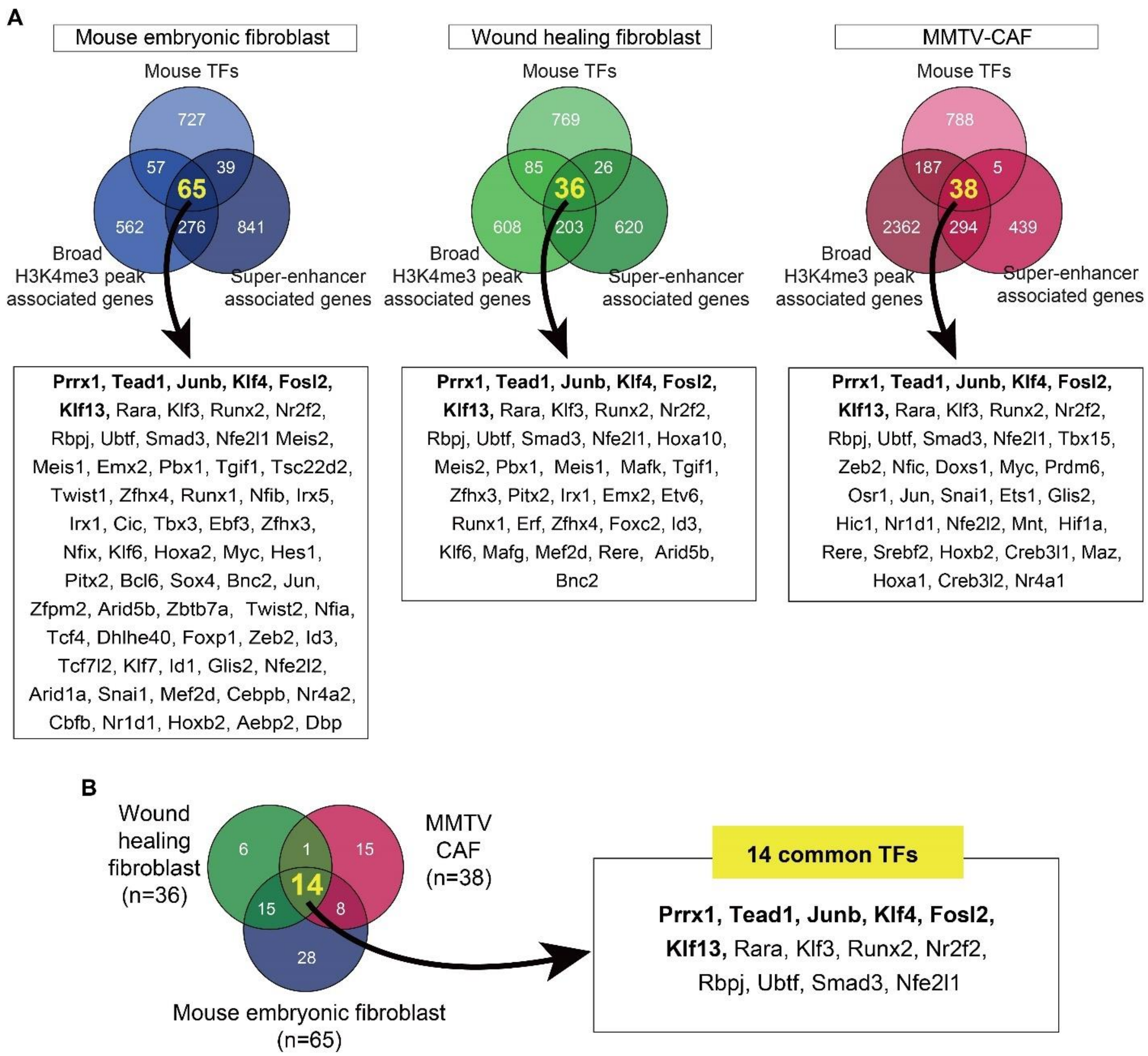

Supplementary Figure 15. Venn diagram of super-enhancer and broad H3K4me3 associated TFs in three types of murine fibroblasts. (A) Venn diagrams show that shared TFs associated with super-enhancer and broad H3K4me3. In mouse embryonic fibroblast (left), wound healing fibroblast (middle), and MMTV-CAF (right), 65, 36, and 38 shared TFs associated with broad H3K4me3 and super-enhancer were identified, respectively. **(B)** 14 TFs were commonly overlapped in three types of murine fibroblasts. Among these TFs, six TRs (Prrx1, Tead1, Junb, Klf4, Fosl2, and Klf13) were included in common CRC of murine activated fibroblasts. (These TFs list related to Figure 4C).

Supplementary Fig. 16

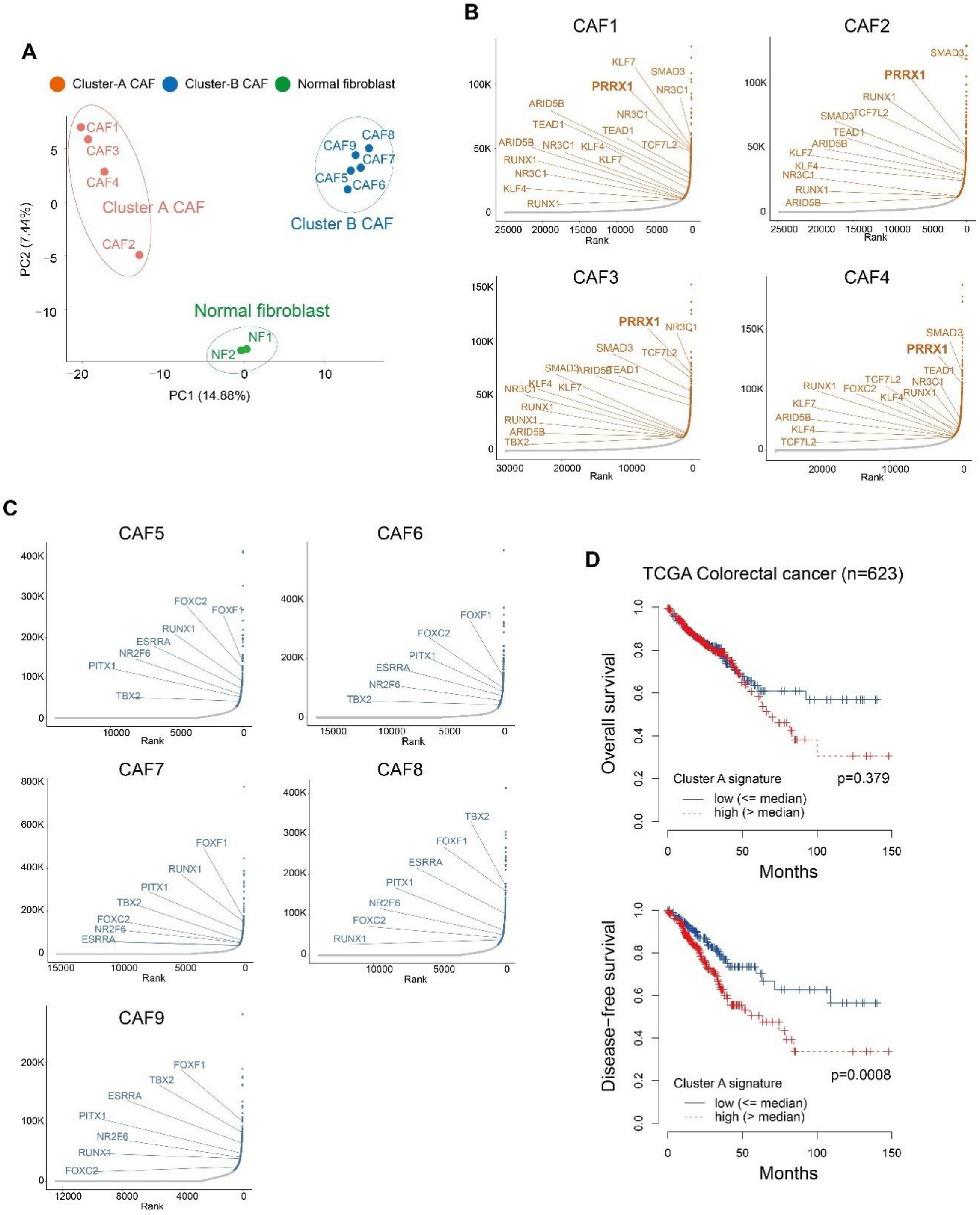

Supplementary Figure 16. Illustration of Super-enhancers of nine human colorectal CAF and normal human colorectal fibroblast. (A) Principal component analysis (PCA) using SEs for the nine colorectal CAFs and normal human colorectal fibroblast discovered three distinct clusters of the colorectal CAFs into the 'Cluster-A' and 'Cluster-B' and normal fibroblast. **(B, C)** Hockey-stick plot shows signal intensity of enhancers. Based on this plot, super-enhnacers were defined (slope was greater than one in the hockey-stick plot) in nine human colorectal CAFs. Identified 'Cluster-A' and 'Cluster-B' master TFs belong to the super-enhancer (Related to Figure 6C right). **(D)** Cluster-A signature was obtained by GSVA using the 463 target genes of SE for Cluster-A group in each bulk RNA-seq data from TCGA colorectal cancer set. The patient group with high "Cluster-A" signature had a poorer prognosis (log-rank test). Source data are provided as a Source Data file.

Supplementary Fig. 17

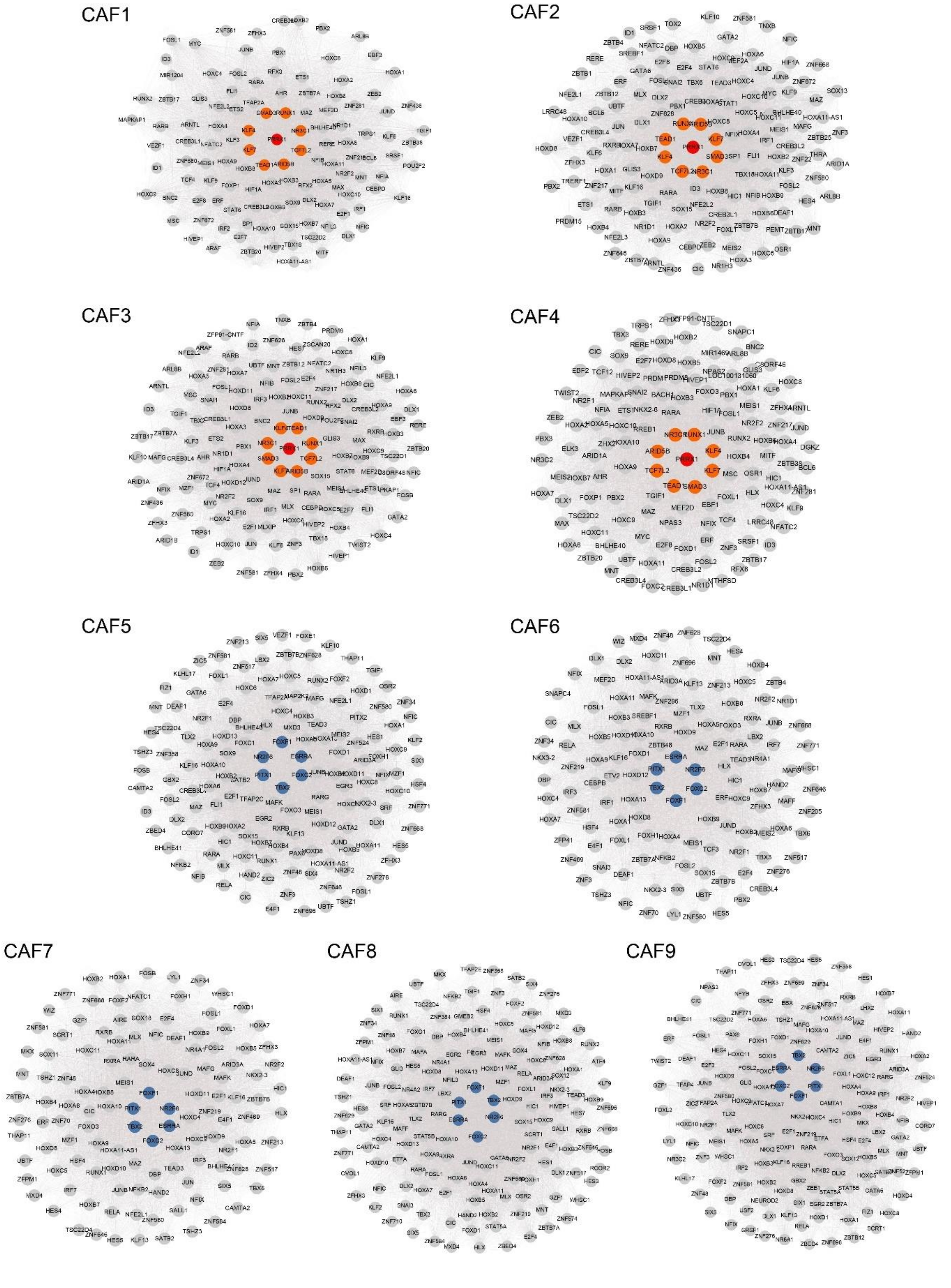

Supplementary Figure 17. Coltron established the Core Regulatory Circuitries (CRCs) of nine colon cancer CAFs to identify TF connectivity. In the center of the extended super-enhancer circuitries, the common CRCs of 'Cluster-A' and 'Cluster-B' CAFs are shown, respectively. In these networks, *PRRX1* appeared in all extended super-enhancer circuitries of only 'Cluster-A' CAFs group. These structures of extended super-enhancer circuitries were used for calculating network structural similarity (see Supplementary Methods for the more details). The calculated scores were shown in Figure 7D.

Supplementary Fig. 18

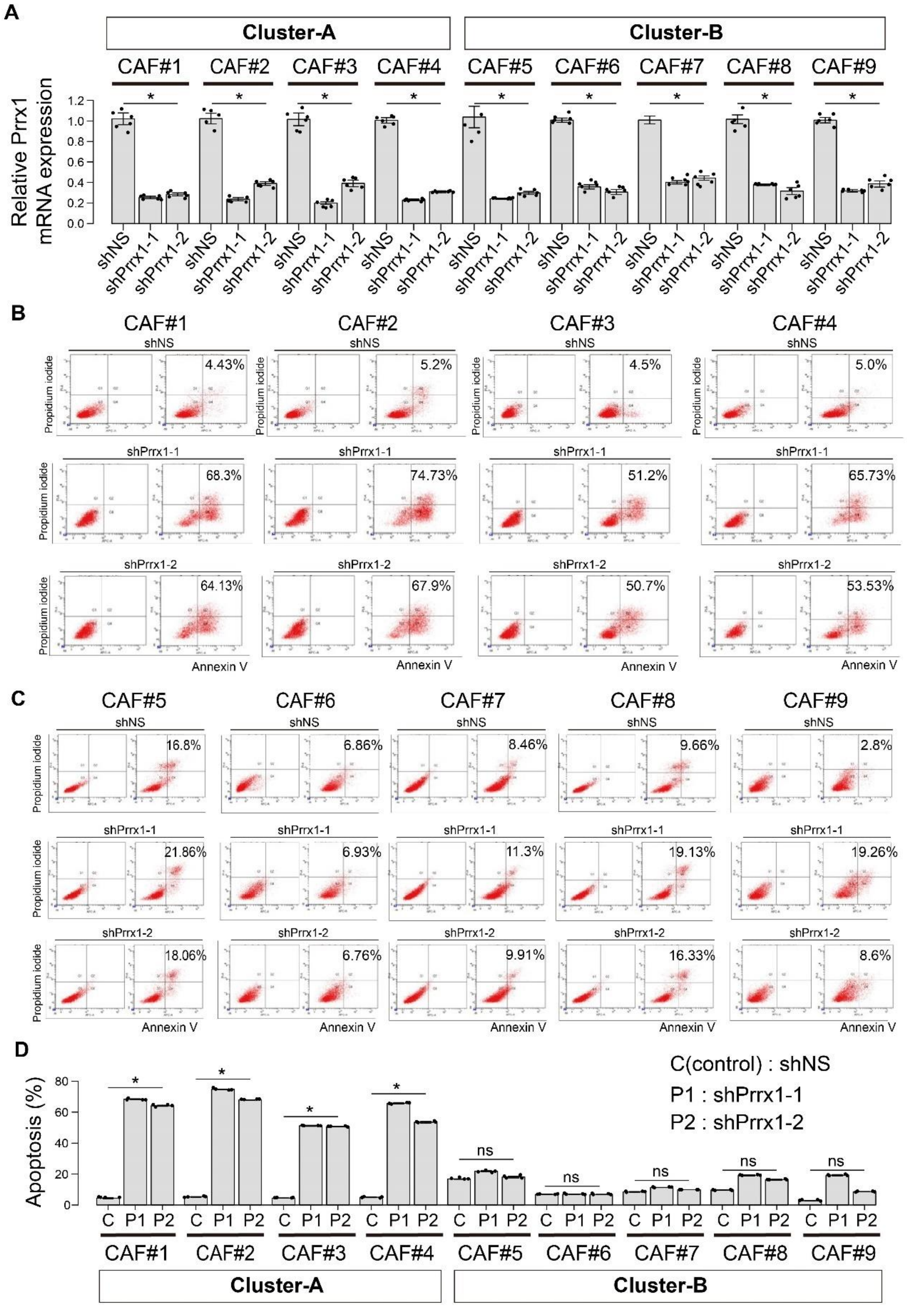

Supplementary Figure 18. Expression of *PRRX1* in Human CAFs and apoptotic capability. **(A)** Confirmation of *PRRX1* mRNA expression level after knockdown of *PRRX1* in 9 CAFs. Data are presented as the mean ± SEM; n =6 independent experiments (*p* value was calculated using two tailed t-test: *p<0.0001). **(B, C)** ShRNA-mediated depletion of *PRRX1* significantly increased massive apoptosis within the initial period (4-5 days after knockdown) in Cluster-A CAFs, but not in Cluster-B CAFs. **(D)** Quantification of the above cell apoptosis assay. The apoptosis assay was performed in 3 replicates. Data are presented as the mean ± SEM; n =3 independent experiments (*p* value was calculated using two tailed t-test: *p<0.0001). Source data and exact p values are provided as a Source Data file.

Supplementary Fig. 19

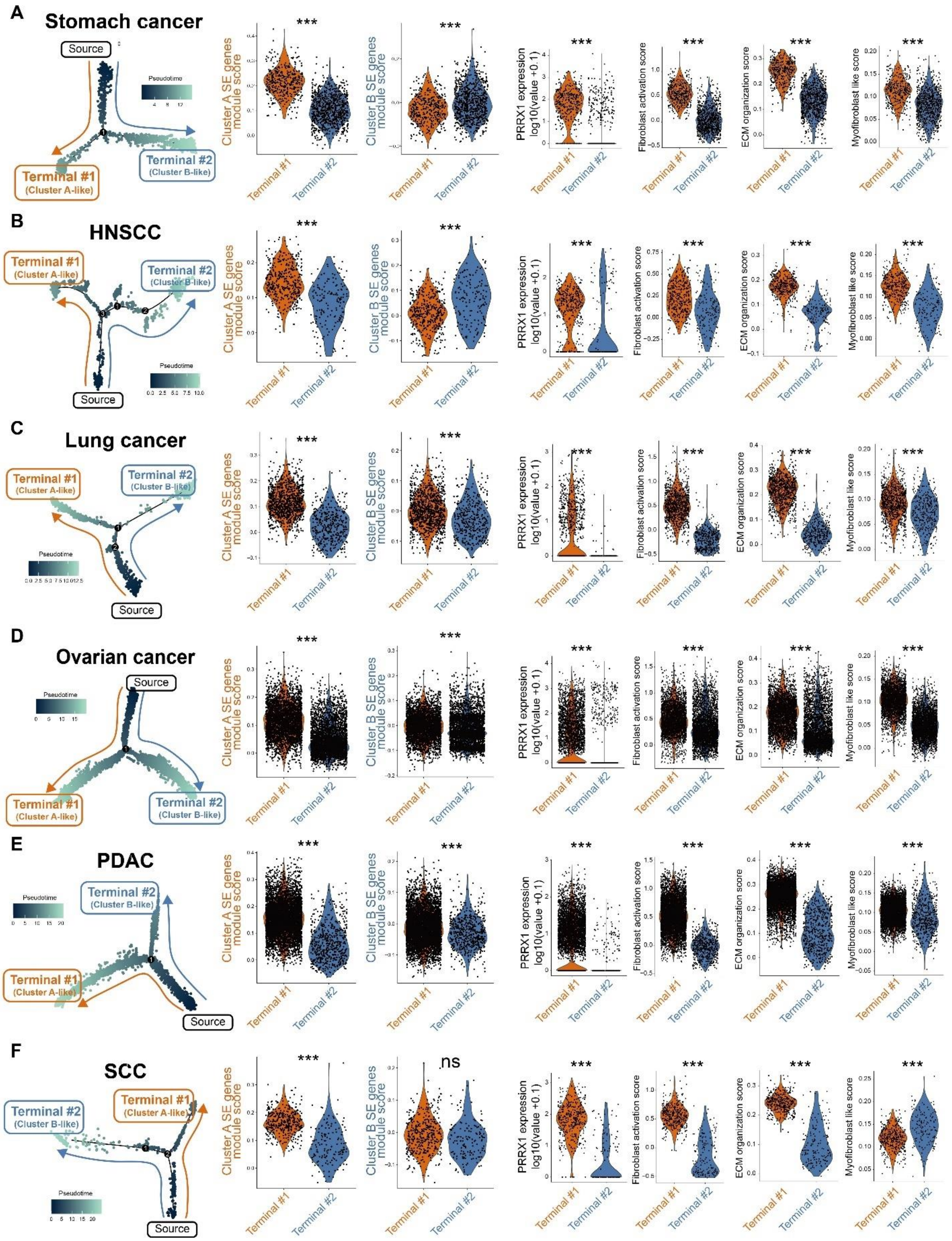

Supplementary Figure 19. Human CAFs with high *PRRX1* expression show different gene expression signatures from those with low *PRRX1* expression (related to Figure. 7I). (A-F) Pseudo-time trajectory analysis of human CAFs from six cancer tissue scRNA-seq data was performed using differentially expressed genes between the high- and low-PRRX1 groups as ordering genes. Each pseudo-time trajectory of human CAFs from six cancer tissues was divided into the two terminal branches (First column). Violin plots show expression levels of genes associated with super-enhancers of Cluster-A and Cluster-B for each terminal in pseudo-time trajectory (Second column). Violin plots of human CAFs from each of six cancer tissues presented four different gene expression signatures between two terminal branches. *PRRX1* expression level is showed as color gradient with logarithmic scale (fourth column). Fibroblast activation score is the average expression level of genes associated with fibroblast activation (fifth column). ECM organization score is the average expression level of genes in Go Extracellular Matrix (sixth column). Myofibroblast-like score is the average expression level of genes in Go Regulation of Supramolecular Fiber Organization (seventh column). The Cluster A-like terminal branch shows higher *PRRX1* expression levels, fibroblast activation score, ECM organization score, and myofibroblast-like score than the Cluster B-like terminal branch. These different gene expression signatures between the two branches support that *PRRX1* is the master TF of human myofibroblastic CAFs (two-sided Wilcoxon rank-sum test, *** $p < 2e\text{-}16$).

Supplementary Fig. 20

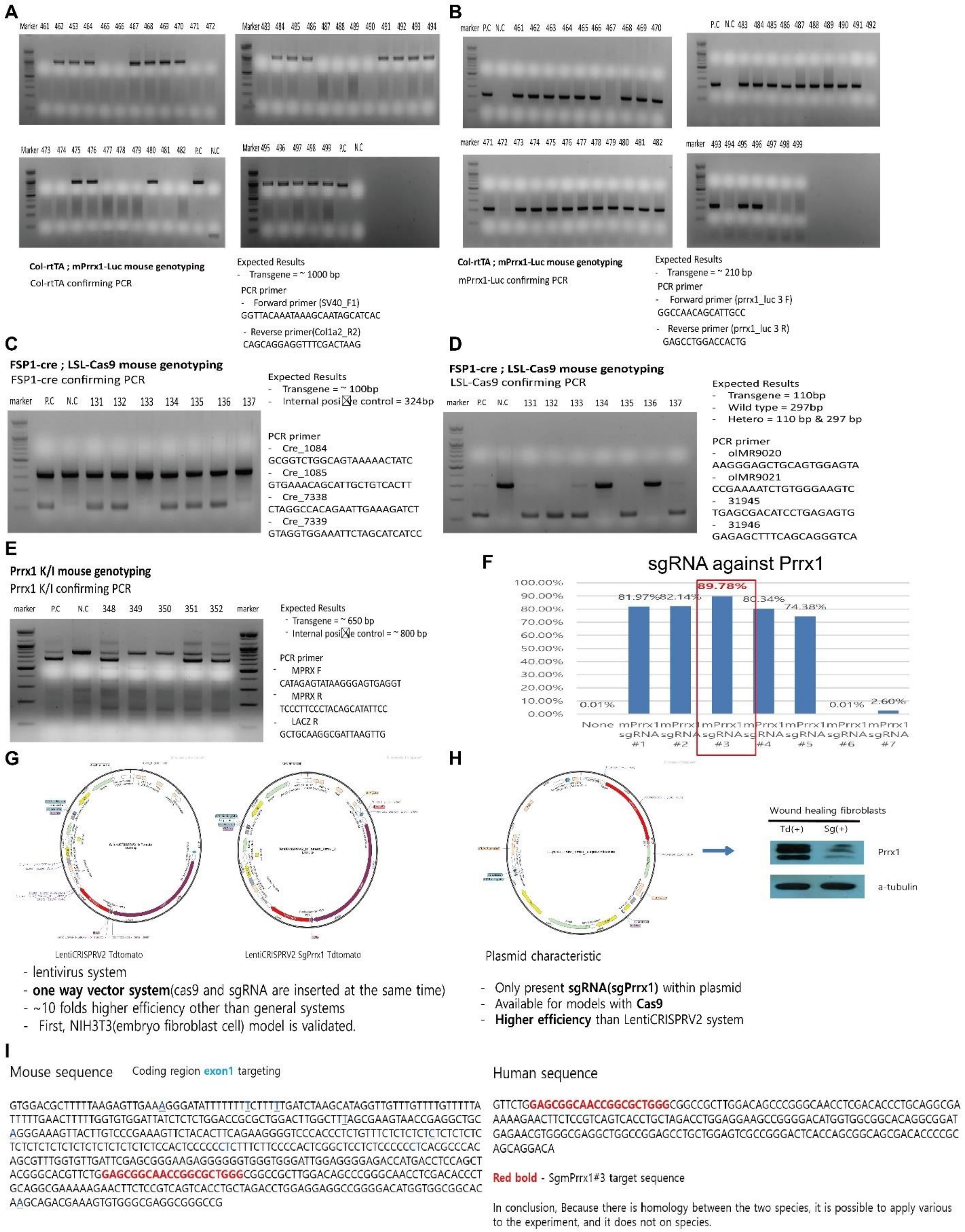

Supplementary Figure 20. Genetic validation of models used for *in vivo* and *in vitro* experiments. (A, B) Representative genotyping gel showing Col1a2^{rtTA};TetO7-Prrx1^{Luc} mice using DNA primers specific for collagen type 1 alpha 2 and Prrx1. Primers and product size are listed below the gel pictures. **(C, D)** Representative genotyping gel showing FSP1^{cre};CAS9^{EGFP} mice using DNA primers specific for Cre recombinase and CAS9. Primers and product size are listed below the gel pictures. **(E)** Prrx1 K/I mice genotyping was performed using DNA primers; specific regions for MPRX F and R are presented before and after, respectively, exon 2 of *Prxx1*. LACZ R is located in the knocked-in LacZ gene. Primers were used to detect both Prrx1 wt (800 bp) and Prrx1 mutant (550 bp). **(F)** Efficiency validation of sgRNA against Prrx1 clones. **(G)** Diagram of the LentiCRISPRV2_Tdtomato and LentiCRISPRV2_sgPrrx1_Tdtomato lentiviral vectors. **(H)** Schematic map of the pLKO.1_Puro_sgPrrx1 plasmid, and validation of Prrx1 efficiency using western blot. **(I)** Generation of plasmids targeting human and mouse Prrx1 exon 1-coding region simultaneously.

Supplementary Fig. 21

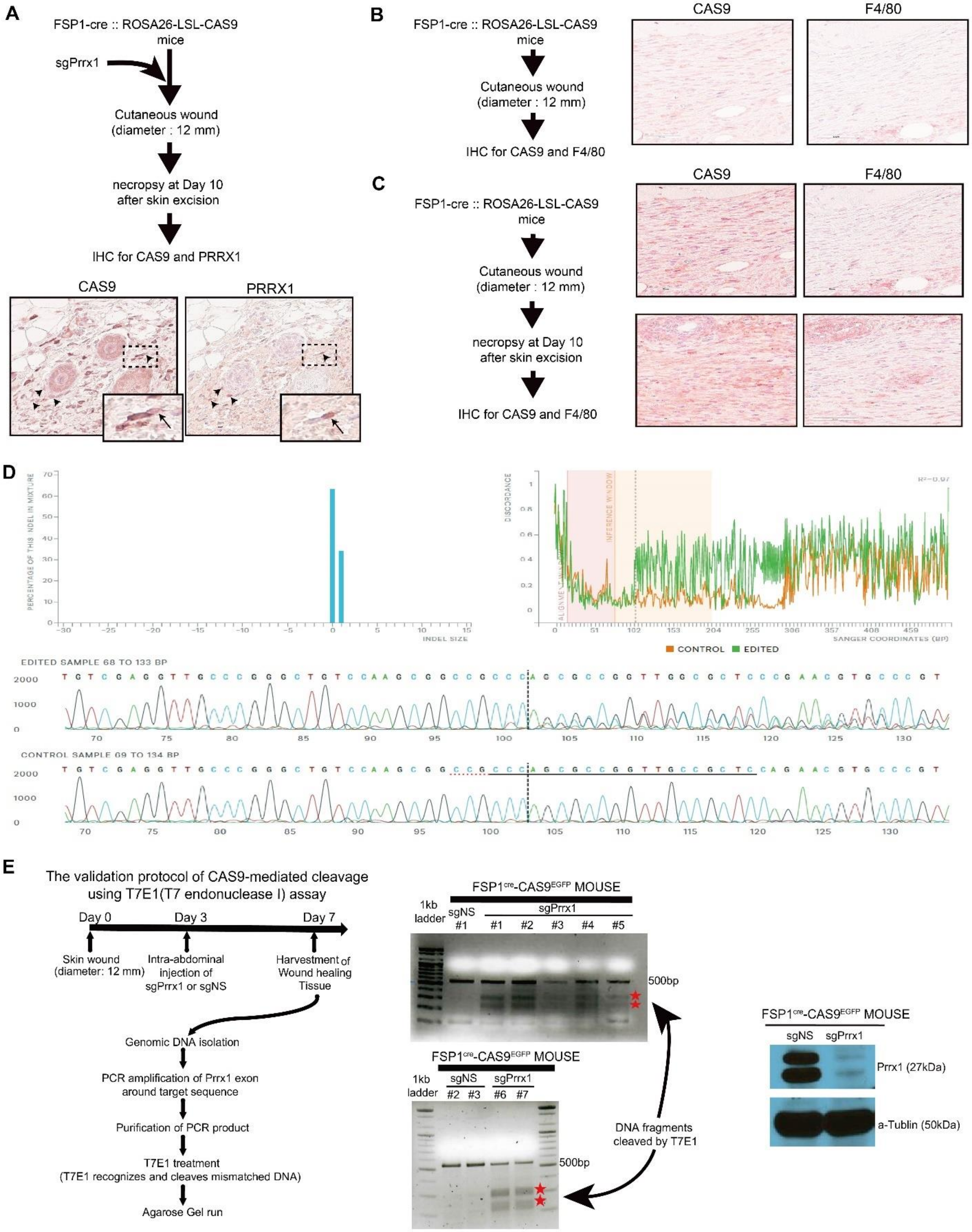

Supplementary Figure 21. Validation for the fibroblast-specific knock-out of Prrx1 in vivo. (A) Schematic illustration of skin wound-healing experiments for tissue processing and immunohistochemical staining images of CAS9 and Prrx1 in each group. **(B, C)** Representative immunohistochemical staining images for CAS9 and F4/80. Images are representative of three tissue samples. **(D)** Synthego ICE analysis of mutations found in each target tissue. Edited sites are shown in the peak graph. **(E)** The validation protocol of CAS9-mediated cleavage using T7E1 assay(sgNS n=3, sgPrrx1 n=7). Representative gel images of T7E1-treated PCR products amplified from target site of Prrx1 exon-1 in each group. Western blotting images of sgRNA-treated tissue. This experiment was independently repeated three times with similar results.

Supplementary Fig. 22

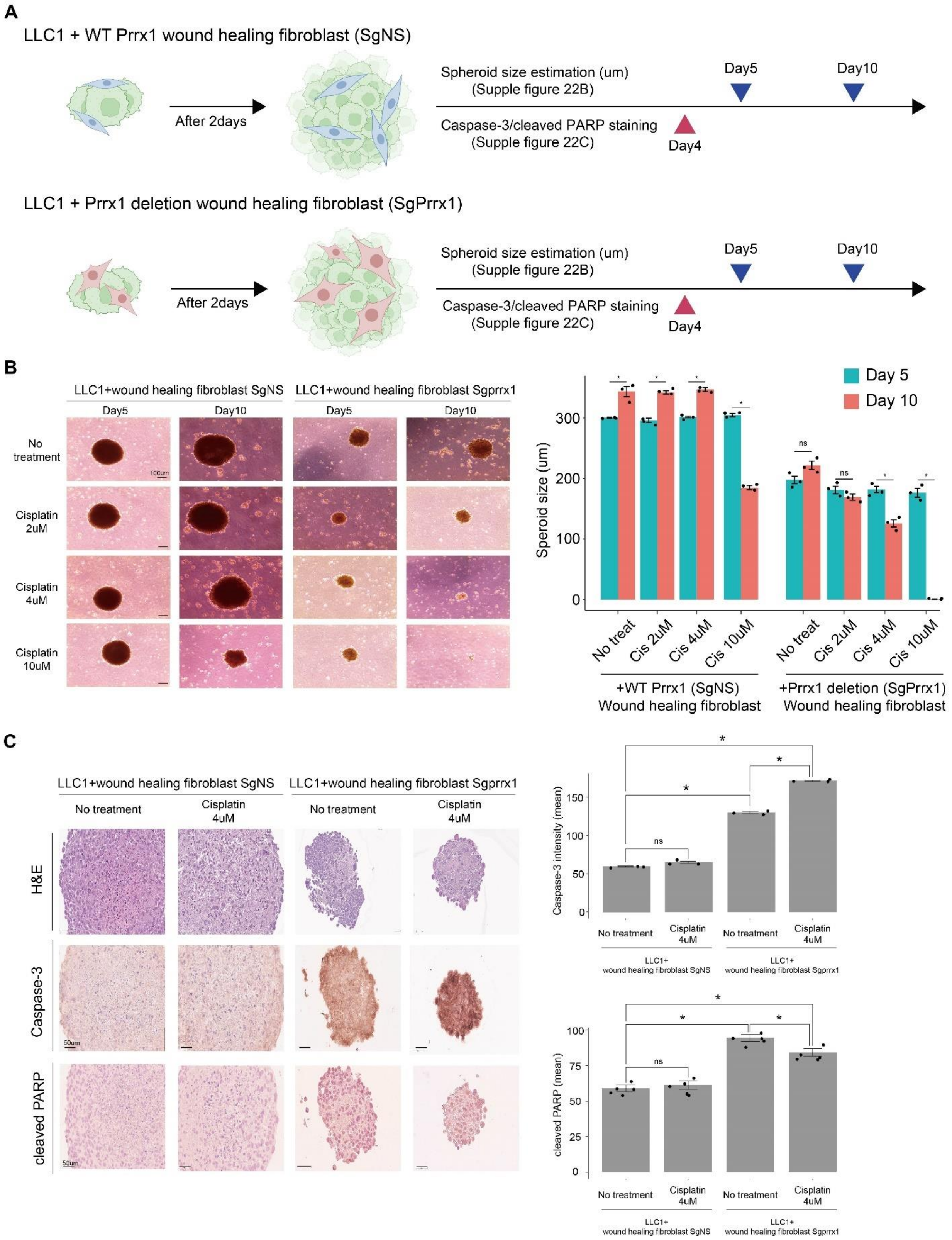

Supplementary Figure 22. Combination treatment of cisplatin with sgPrrx1 induced fibroblast specific Prrx1 deletion resulted in tumor cell death in an in vitro co-culture system. (A) Establishment of a hanging drop direct 3D spheroid co-culture system to mimic the in vivo mouse model used in Figure 9. **(B)** Combination treatment of cisplatin and sgPrrx1 resulted in inhibition of spheroid growth. (n =3 independent measurements, *p* value was calculated using two tailed t-test: *p<0.0001). **(C)** Representative H&E images of spheroids of each group and immunohistochemical staining images for caspase3. Combination treatment of cisplatin and sgPrrx1 resulted in massive cell death, suggesting a certain synergistic effect between them. Quantification of caspase3 and cleaved-PARP intensity (mean) was determined using ImageJ Fiji. (n =3 independent measurements, *p* value was calculated using two tailed t-test: *p<0.05). Source data and exact p values are provided as a Source Data file.

Supplementary References

1 Akiyama, K. *et al.* Characterization of bone marrow derived mesenchymal stem cells in suspension. *Stem cell research & therapy* **3**, 40, doi:10.1186/scrt131 (2012).

2 Lambrechts, D. *et al.* Phenotype molding of stromal cells in the lung tumor microenvironment. *Nature medicine* **24**, 1277-1289, doi:10.1038/s41591-018-0096-5 (2018).

3 Qian, J. *et al.* A pan-cancer blueprint of the heterogeneous tumor microenvironment revealed by single-cell profiling. *Cell research* **30**, 745-762, doi:10.1038/s41422-020-0355-0 (2020).

4 Ji, A. L. *et al.* Multimodal Analysis of Composition and Spatial Architecture in Human Squamous Cell Carcinoma. *Cell* **182**, 497-514.e422, doi:10.1016/j.cell.2020.05.039 (2020).

5 Sathe, A. *et al.* Single-Cell Genomic Characterization Reveals the Cellular Reprogramming of the Gastric Tumor Microenvironment. *Clinical cancer research : an official journal of the American Association for Cancer Research* **26**, 2640-2653, doi:10.1158/1078-0432.Ccr-19-3231 (2020).

6 Peng, J. *et al.* Single-cell RNA-seq highlights intra-tumoral heterogeneity and malignant progression in pancreatic ductal adenocarcinoma. *Cell research* **29**, 725-738, doi:10.1038/s41422-019-0195-y (2019).

7 Puram, S. V. *et al.* Single-Cell Transcriptomic Analysis of Primary and Metastatic Tumor Ecosystems in Head and Neck Cancer. *Cell* **171**, 1611-1624.e1624, doi:10.1016/j.cell.2017.10.044 (2017).

8 Zhao, T. *et al.* Single-Cell RNA-Seq Reveals Dynamic Early Embryonic-like Programs during Chemical Reprogramming. *Cell stem cell* **23**, 31-45.e37, doi:10.1016/j.stem.2018.05.025 (2018).

9 Guerrero-Juarez, C. F. *et al.* Single-cell analysis reveals fibroblast heterogeneity and myeloid-derived adipocyte progenitors in murine skin wounds. *Nature communications* **10**, 650, doi:10.1038/s41467-018-08247-x (2019).

10 Hosein, A. N. *et al.* Cellular heterogeneity during mouse pancreatic ductal adenocarcinoma progression at single-cell resolution. *JCI insight* **5**, doi:10.1172/jci.insight.129212 (2019).

11 Yeo, S. Y. *et al.* A positive feedback loop bi-stably activates fibroblasts. *Nature communications* **9**, 3016, doi:10.1038/s41467-018-05274-6 (2018).

12 Bolger, A. M., Lohse, M. & Usadel, B. Trimmomatic: a flexible trimmer for Illumina sequence data. *Bioinformatics* **30**, 2114-2120, doi:10.1093/bioinformatics/btu170 (2014).

13 Langmead, B., Trapnell, C., Pop, M. & Salzberg, S. L. Ultrafast and memory-efficient alignment of short DNA sequences to the human genome. *Genome biology* **10**, R25, doi:10.1186/gb-2009-10-3-r25 (2009).

14 Zhang, Y. *et al.* Model-based analysis of ChIP-Seq (MACS). *Genome biology* **9**, R137, doi:10.1186/gb-2008-9-9-r137 (2008).
15 Whyte, W. A. *et al.* Master transcription factors and mediator establish super-enhancers at key cell identity genes. *Cell* **153**, 307-319, doi:10.1016/j.cell.2013.03.035 (2013).
16 Lin, C. Y. *et al.* Active medulloblastoma enhancers reveal subgroup-specific cellular origins. *Nature* **530**, 57-62, doi:10.1038/nature16546 (2016).
17 Li, H. *et al.* The Sequence Alignment/Map format and SAMtools. *Bioinformatics* **25**, 2078-2079, doi:10.1093/bioinformatics/btp352 (2009).
18 Quinlan, A. R. & Hall, I. M. BEDTools: a flexible suite of utilities for comparing genomic features. *Bioinformatics* **26**, 841-842, doi:10.1093/bioinformatics/btq033 (2010).
19 Heinz, S. *et al.* Simple combinations of lineage-determining transcription factors prime cis-regulatory elements required for macrophage and B cell identities. *Molecular cell* **38**, 576-589, doi:10.1016/j.molcel.2010.05.004 (2010).
20 Robinson, J. T. *et al.* Integrative genomics viewer. *Nat Biotechnol* **29**, 24-26, doi:10.1038/nbt.1754 (2011).
21 Ramírez, F. *et al.* deepTools2: a next generation web server for deep-sequencing data analysis. *Nucleic acids research* **44**, W160-165, doi:10.1093/nar/gkw257 (2016).
22 Kuleshov, M. V. *et al.* Enrichr: a comprehensive gene set enrichment analysis web server 2016 update. *Nucleic acids research* **44**, W90-97, doi:10.1093/nar/gkw377 (2016).
23 Ross-Innes, C. S. *et al.* Differential oestrogen receptor binding is associated with clinical outcome in breast cancer. *Nature* **481**, 389-393, doi:10.1038/nature10730 (2012).
24 Dobin, A. *et al.* STAR: ultrafast universal RNA-seq aligner. *Bioinformatics* **29**, 15-21, doi:10.1093/bioinformatics/bts635 (2013).
25 Anders, S., Pyl, P. T. & Huber, W. HTSeq--a Python framework to work with high-throughput sequencing data. *Bioinformatics* **31**, 166-169, doi:10.1093/bioinformatics/btu638 (2015).
26 Sergushichev, A. A. An algorithm for fast preranked gene set enrichment analysis using cumulative statistic calculation. 060012, doi:10.1101/060012 %J bioRxiv (2016).
27 Hänzelmann, S., Castelo, R. & Guinney, J. GSVA: gene set variation analysis for microarray and RNA-seq data. *BMC bioinformatics* **14**, 7, doi:10.1186/1471-2105-14-7 (2013).
28 Zheng, G. X. *et al.* Massively parallel digital transcriptional profiling of single cells. *Nature communications* **8**, 14049, doi:10.1038/ncomms14049 (2017).
29 Hao, Y. *et al.* Integrated analysis of multimodal single-cell data. *Cell* **184**, 3573-3587.e3529, doi:10.1016/j.cell.2021.04.048 (2021).
30 Zhang, A. W. *et al.* Probabilistic cell-type assignment of single-cell RNA-seq for tumor microenvironment profiling. *Nature methods* **16**, 1007-1015, doi:10.1038/s41592-019-0529-1 (2019).

31 Browaeys, R., Saelens, W. & Saeys, Y. NicheNet: modeling intercellular communication by linking ligands to target genes. *Nature methods* **17**, 159-162, doi:10.1038/s41592-019-0667-5 (2020).

32 Love, M. I., Huber, W. & Anders, S. Moderated estimation of fold change and dispersion for RNA-seq data with DESeq2. *Genome biology* **15**, 550, doi:10.1186/s13059-014-0550-8 (2014).

33 Stuart, T. *et al.* Comprehensive Integration of Single-Cell Data. *Cell* **177**, 1888-1902.e1821, doi:10.1016/j.cell.2019.05.031 (2019).

34 Racle, J., de Jonge, K., Baumgaertner, P., Speiser, D. E. & Gfeller, D. Simultaneous enumeration of cancer and immune cell types from bulk tumor gene expression data. *eLife* **6**, doi:10.7554/eLife.26476 (2017).

35 Qiu, X. *et al.* Single-cell mRNA quantification and differential analysis with Census. *Nature methods* **14**, 309-315, doi:10.1038/nmeth.4150 (2017).

36 Mahmoudi, S. *et al.* Heterogeneity in old fibroblasts is linked to variability in reprogramming and wound healing. *Nature* **574**, 553-558, doi:10.1038/s41586-019-1658-5 (2019).

37 Liberzon, A. *et al.* Molecular signatures database (MSigDB) 3.0. *Bioinformatics* **27**, 1739-1740, doi:10.1093/bioinformatics/btr260 (2011).

38 Gao, R. *et al.* Delineating copy number and clonal substructure in human tumors from single-cell transcriptomes. *Nat Biotechnol* **39**, 599-608, doi:10.1038/s41587-020-00795-2 (2021).

39 Zhang, Y., Parmigiani, G. & Johnson, W. E. ComBat-seq: batch effect adjustment for RNA-seq count data. *NAR genomics and bioinformatics* **2**, lqaa078, doi:10.1093/nargab/lqaa078 (2020).

40 Hothorn, T., Lausen, B. J. C. S. & Analysis, D. On the exact distribution of maximally selected rank statistics. **43**, 121-137 (2003).
