# Supplementary material for: PRRX1 is a master transcription factor of stromal fibroblasts for myofibroblastic lineage progression
Source: Nat Commun. 2022 May 19;13:2793. doi: 10.1038/s41467-022-30484-4 (PMC9120014; doi:10.1038/s41467-022-30484-4)
Supplement: Supplementary file 2 — Description of Additional Supplementary Files [file 41467_2022_30484_MOESM2_ESM.pdf]

Description of Additional Supplementary Files

File Name: Supplementary Data 1
Description: Associations between Prrx1 expression and clinicopathologic variables in three cancer tissues. The high Prrx1 expression levels in three types of cancer tissue (colon, stomach, and esophageal cancer) were significantly correlated with advanced TNM stage, advanced T classification, and tumor size.

File Name: Supplementary Data 2
Description: Enhancers and super-enhancers from three types of murine fibroblasts and nine human cancer-associated fibroblasts.

File Name: Supplementary Data 3
Description: Correlation of ATAC-seq (Assay for Transposase-Accesible Chromatin sequencing) and Chip-seq in nine CAFs.

File Name: Supplementary Data 4

Description: Transcription factors (TFs) that make up core regulatory circuits (CRCs) originated from three types of murine fibroblasts and nine human CAFs.
