# Supplementary material for: PRRX1 is a master transcription factor of stromal fibroblasts for myofibroblastic lineage progression
Source: Nat Commun. 2022 May 19;13:2793. doi: 10.1038/s41467-022-30484-4 (PMC9120014; doi:10.1038/s41467-022-30484-4)
Supplement: Supplementary file 8 — Reporting Summary [file 41467_2022_30484_MOESM8_ESM.pdf]

nature research

Corresponding author(s): Seok-Hyung kim; Kwang-Hyun Cho; Chang Ohk Sung

Last updated by author(s): 05/April/22

Reporting Summary

Nature Research wishes to improve the reproducibility of the work that we publish. This form provides structure for consistency and transparency in reporting. For further information on Nature Research policies, see our Editorial Policies and the Editorial Policy Checklist.

Statistics

For all statistical analyses, confirm that the following items are present in the figure legend, table legend, main text, or Methods section.

n/a	Confirmed	
☐	☒	The exact sample size (*n*) for each experimental group/condition, given as a discrete number and unit of measurement
☐	☒	A statement on whether measurements were taken from distinct samples or whether the same sample was measured repeatedly
☐	☒	The statistical test(s) used AND whether they are one- or two-sided<br>*Only common tests should be described solely by name; describe more complex techniques in the Methods section.*
☐	☒	A description of all covariates tested
☒	☐	A description of any assumptions or corrections, such as tests of normality and adjustment for multiple comparisons
☐	☒	A full description of the statistical parameters including central tendency (e.g. means) or other basic estimates (e.g. regression coefficient) AND variation (e.g. standard deviation) or associated estimates of uncertainty (e.g. confidence intervals)
☒	☐	For null hypothesis testing, the test statistic (e.g. *F*, *t*, *r*) with confidence intervals, effect sizes, degrees of freedom and *P* value noted<br>*Give P values as exact values whenever suitable.*
☒	☐	For Bayesian analysis, information on the choice of priors and Markov chain Monte Carlo settings
☒	☐	For hierarchical and complex designs, identification of the appropriate level for tests and full reporting of outcomes
☒	☐	Estimates of effect sizes (e.g. Cohen's *d*, Pearson's *r*), indicating how they were calculated

Our web collection on statistics for biologists contains articles on many of the points above.

Software and code

Policy information about availability of computer code

Data collection	No software was used to data collection.
Data analysis	Trimmomatic v0.39 Bolger et. al., 2014 http://www.usadellab.org/cms/index.php?page=trimmomatic<br>Bowtie v1.3.0 Langmead et. al., 2009 http://bowtie-bio.sourceforge.net/index.shtml<br>MACS2 Zhang et. al., 2008 https://github.com/macs3-project/MACS<br>ROSE Whyte et. al., 2013 http://younglab.wi.mit.edu/super_enhancer_code.html<br>Coltron Lin et. al., 2016 https://pypi.org/project/coltron/<br>Samtools v1.7 Li et. al., 2009 https://github.com/samtools/<br>Bedtools v2.28.0 Quinlan et. al., 2010 https://github.com/arq5x/bedtools2<br>Homer Heinz et. al., 2010 http://homer.ucsd.edu/homer/<br>Integrative Genomics Viewer Robinson et. al., 2011 http://software.broadinstitute.org/software/igv/<br>Deeptools v3.3.1 Ramírez et. al., 2016 https://github.com/deeptools/deepTools/blob/develop/docs/index.rst<br>EnrichR Kuleshov et. al., 2016 https://cran.r-project.org/web/packages/enrichR/index.html<br>DiffBind Ross-Innes et. al., 2012 https://www.bioconductor.org/packages/release/bioc/html/DiffBind.html<br>STAR v2.7.7a Dobin et. al., 2013 https://github.com/alexdobin/STAR<br>HTSeq v0.12.4 Anders et. al., 2014 https://htseq.readthedocs.io/en/master/overview.html<br>fgsea Sergushichev et. al., 2019 https://bioconductor.org/packages/release/bioc/html/fgsea.html<br>GSVA Hänzelmann et. al., 2013 https://www.bioconductor.org/packages/release/bioc/html/GSVA.html<br>Cellranger v3.1.0 Zheng et. al., 2017 https://support.10xgenomics.com/single-cell-gene-expression/<br>Seurat v4.0.0 Hao et. al., 2020 https://satijalab.org/seurat/index.html<br>CellAssigner Zhang et. al., 2019 https://github.com/Irrationone/cellassign<br>NichenetR Browaeys et. al., 2020 https://github.com/saeyslab/nichenetr

DEseq2 Love et. al., 2014 https://bioconductor.org/packages/release/bioc/html/DESeq2.html
R 4.0.3 N/A https://www.r-project.org/

For manuscripts utilizing custom algorithms or software that are central to the research but not yet described in published literature, software must be made available to editors and reviewers. We strongly encourage code deposition in a community repository (e.g. GitHub). See the Nature Research guidelines for submitting code & software for further information.

Data

Policy information about availability of data

All manuscripts must include a data availability statement. This statement should provide the following information, where applicable:

- Accession codes, unique identifiers, or web links for publicly available datasets
- A list of figures that have associated raw data
- A description of any restrictions on data availability

The accession number for ChIP, RNA, and single cell RNA sequencing data reported in this paper are available on the NCBI Gene Expression Omnibus (GEO) database under the accession codes such as GSE169725(Super-series), GSE169703 (Three types of murine fibroblasts RNA-seq), GSE169723 (Three types of murine fibroblasts ChIP-seq), GSE169697 (RNA-seq data of Co-cultures of cancer spheroid with fibroblasts), GSE169720 (Human cancer associated fibroblasts RNA-seq), GSE169601 (Human cancer associated fibroblasts ChIP-seq), GSE169704 (Single cell RNA-seq from murine wound tissue). The authors declare that all the data supporting the results of this study are available within the article and its supplementary information files and from the corresponding authors upon reasonable request.

Field-specific reporting

Please select the one below that is the best fit for your research. If you are not sure, read the appropriate sections before making your selection.

[x] Life sciences [ ] Behavioural & social sciences [ ] Ecological, evolutionary & environmental sciences

For a reference copy of the document with all sections, see nature.com/documents/nr-reporting-summary-flat.pdf

Life sciences study design

All studies must disclose on these points even when the disclosure is negative.

Sample size	We used total 21 cell lines or organisms. This number of samples was sufficient because the results were verified through different types of repeated experiments on a variety of different cell lines. Patients cancer tissues for five cancer types such as colorectal cancer (n=185), stomach cancer (n=178), lung cancer (n=80), esophageal cancer (n=168), and breast cancer (n=80) were also sufficient to obtained statistical significance in survival analysis becasue most of the survival data for long-term follow-up were obtained. Sample size is indicated in the figure legend for each experiments. For cell-based qunatitative experiments, results of three independent biological replicates were used. In mouse experiments, we used mouse numbers that are sufficient for calculation. The group size is more than 5 animals/group for an each experiment.
Data exclusions	No data were exclued from the analyses.
Replication	All attempts at replication were successful. We have included detailed numbers in the manuscript.
Randomization	Samples were allocated as random. In mouse experiments, Mice were randomized into groups of equal average tumor volume to the treatment groups.
Blinding	Investigators were blinded to group allocation during the analysis. No other experiments required collection or quantification to be blinded.

Reporting for specific materials, systems and methods

We require information from authors about some types of materials, experimental systems and methods used in many studies. Here, indicate whether each material, system or method listed is relevant to your study. If you are not sure if a list item applies to your research, read the appropriate section before selecting a response.

Materials & experimental systems

n/a	Involved in the study	
[ ]	[x]	Antibodies
[ ]	[x]	Eukaryotic cell lines
[x]	[ ]	Palaeontology and archaeology
[ ]	[x]	Animals and other organisms
[ ]	[x]	Human research participants
[ ]	[x]	Clinical data
[x]	[ ]	Dual use research of concern

Methods

n/a	Involved in the study	
[ ]	[x]	ChIP-seq
[ ]	[x]	Flow cytometry
[x]	[ ]	MRI-based neuroimaging

Antibodies

Antibodies used	Antibodies for IHC<br>Anti-Prrx1 (Origene Cat# TA803116, RRID:AB_2620166, 1:400)<br>Anti-PRRX1 (LS-bio Cat# LS-C336798, RRID: AB_2725124, 1:500)<br>Anti-aSMA (DAKO Cat# M0851; RRID:AB_2313736, 1:200)<br>Anti-PCNA (Abcam Cat# ab29, RRID:AB_303394, 1:1000)<br>Anti-Tenascin C (Abcam Cat# ab108930, RRID:AB_10865908, 1:100)<br>Anti-PDGFRa (Cell signaling Cat# 3164, RRID:AB_2162351, 1:100)<br>Anti- Cas9 (Cell signaling Cat# 14697S, RRID:AB_2750916, 1:300)<br>Anti- F4/80 (Cell signaling Cat# 70076S, RRID:AB_2799771, 1:500)<br>Anti-Caspase-3 (Cell signaling Cat# 14220, RRID:AB_2798429, 1:300)<br>Anti-Cleaved PARP (Cell signaling Cat# 5625, RRID:AB_10699460, 1:300)<br><br>Antibodies for ChIP-seq:<br>Anti-H3K27ac (Abcam Cat# ab4729, RRID:AB_2118291, 5ug per test)<br>Anti-Histone H3 (tri methyl K4) (Abcam Cat# ab8580, RRID:AB_306649, 5ug per test)<br>Anti-normal mouse IgG (Santa Cruz Biotechnology Cat# sc-2025, RRID:AB_737182, 5ug per test)<br><br>Antibodies for western blot:<br>Anti-Prrx1 (Origene Cat# TA803116,RRID:AB_2620166, 1:1000)<br>Anti-a-Tubulin (Santa Cruz Cat# sc-8035, RRID:AB_628408, 1:3000)<br>Anti-SMAD3 (Cell signaling Cat# 9523, RRID:AB_10860597, 1:1000)
Validation	All antibodies were validated using positive and negative controls and/or western blot and used in this study are commercially available and have been verified by the manufacturers according to the data on their websites.

Eukaryotic cell lines

Policy information about cell lines

Cell line source(s)	HT29, HCT116, SNU668, MKN28 cancer cells were from Korea Cell Line Bank (KCLB). 168FARN cell was a gift from Dr. Jing Yang (Cell. 2004 Jun 25;117(7):927-39). LLC1 cell was obtained from the American Type Culture Collection (ATCC). MC38 cell was provided by Dr. Hyon E. Choy (Oncotarget. 2018 Jan 5;9(9):8548-8559). LLC1-GFP, 168FARN-GFP and MC38-GFP were home-made.
Authentication	For all cell lines used in the manuscript, cell line identity was authenticated by supplier with short tandem repeat profiling .
Mycoplasma contamination	All cells lines used were negative for mycoplasma. Cell lines have been tested by using the Mycoplasma Detection kit for conventionnal PCR (Biological Industries, EZ-PCR Mycoplasma Test Kit #20-700-20).
Commonly misidentified lines<br>(See ICLAC register)	MKN28: MKN28 cell line was reported as gastric tubular adenocarcinoma cell line established from 70-year-old female patient. Recently JCRB cell bank found by a DNA analysis that MKN28 is the derivative of MKN74 (JCRB0255) that was established from 37-year-old male. MKN74 was also derived from gastric tubular adenocarcinoma. JCRB cell bank examined several lots of MKN28 that were preserved independently in other organizations in Japan, and found all lots examined exhibit the same DNA pattern as MKN74. Therefore, MKN28 cell lines widely used is, in fact, likely to be the contaminant of MKN74. In our lab, we purchased this cell line for gastric cancer from Korea Cell Line Bank before announcement of this issue in the Cell Line Bank. However we didn't use MKN74 in this study.

Animals and other organisms

Policy information about studies involving animals; ARRIVE guidelines recommended for reporting animal research

Laboratory animals	Tumor studies involving transgenic mice included males and females (Mus musculus) on a C57BL6 background aged 1 to 4 months and other stains as described in the Methods section.<br><br>Mouse: MMTV-PyMT (backcrossed to FVB/N) Jackson Laboratory RRID:IMSR_GPT:T004993<br>Mouse: Prrx1tm1Jfm/Mmmh (backcrossed to C57BL6) MMRRC RRID:MMRRC_000347-MU<br>Mouse: S100a4-cre (backcrossed to BALB/c) Jackson Laboratory IMSR Cat# JAX:012641, RRID:IMSR_JAX:012641<br>Mouse: Rosa26-LSL-Cas9 knockin (backcrossed to C57BL6) Jackson Laboratory RRID:IMSR_JAX:024857<br>Mouse: C57BL/6J Jackson Laboratory RRID:IMSR_JAX:000664<br>Mouse: NSG (NOD.Cg-Prkdcscid Il2rgtm1Wjl/SzJ) Jackson Laboratory RRID:IMSR_NM-NSG-012 Stock No: 005557<br>Mouse: NOD/SCID Jackson Laboratory RRID:IMSR_JAX:001303<br>Mouse: Col1a2-rtTA (backcrossed to C57BL6) This paper N/A<br>Mouse: TetO7-Prrx1-Luciferase (backcrossed to C57BL6) This paper N/A<br><br>Mice were housed in the ventilated cage (max 5 mice/cage) supplied with food and water in a 12-h light/12-h dark cycle at 22°C and 41% humidity.

Wild animals	The study did not involve wild animals.
Field-collected samples	The study did not involve samples collected from the field.
Ethics oversight	All studies and procedures involving animal subjects were approved by the institutional Animal Care and Use Committee (IACUC) of Laboratory Animal Research Center at Samsung Biomedical Research Institute (Protocol #20150424001, 20190607001, 20191017002, 2021012004, 20210317001). Animal Facility was provided by the Association for Assessment and Accreditation of Laboratory Animal Care (AAALAC).

Note that full information on the approval of the study protocol must also be provided in the manuscript.

Human research participants

Policy information about studies involving human research participants

Population characteristics	Tissue samples were prepared from patients with five cancer tissues including colorectal cancer (n=185), stomach cancer (n=178), lung cancer (n=80), esophageal cancer (n=168), and breast cancer (n=80) who confirmed as pathologic diagnosis. For the isolation of cancer associated fibroblasts and Normal fibroblasts from fresh colon cancer patient (n=9) undergoing surgery at Samsung Medical Center.
Recruitment	The samples were collected as standard of care. The samples were selected as randomly to minimize selection biases.
Ethics oversight	The study protocol was approved by the institutional review board (SMC 2021-02-048) of Samsung Medical Center (Seoul, Korea).

Note that full information on the approval of the study protocol must also be provided in the manuscript.

Clinical data

Policy information about clinical studies

All manuscripts should comply with the ICMJEguidelines for publication of clinical research and a completedCONSORT checklist must be included with all submissions.

Clinical trial registration	No samples were involved in clinical trials.
Study protocol	No samples were involved in clinical trials.
Data collection	No samples were involved in clinical trials.
Outcomes	No samples were involved in clinical trials.

ChIP-seq

Data deposition

- [x] Confirm that both raw and final processed data have been deposited in a public database such as GEO.
- [x] Confirm that you have deposited or provided access to graph files (e.g. BED files) for the called peaks.

Data access links<br>*May remain private before publication.*	https://www.ncbi.nlm.nih.gov/geo/<br>GSE169723<br>GSE169601
Files in database submission	BW, BED, and BAM files
Genome browser session<br>(e.g. UCSC)	Not applicable.

Methodology

Replicates	Three types of mouse fibroblasts and nine human CAFs were experimented.
Sequencing depth	All ChiP-seq experiments were performed in same batch. We have obtained average 82134896 x 2 of total number of reads by paired end sequencing with 101 bp of read length. We obtained more than 80% of unique mapping rate.
Antibodies	Anti-H3K27ac Abcam Cat# ab4729, RRID:AB_2118291<br>Anti-Histone H3 (tri methyl K4) Abcam Cat# ab8580, RRID:AB_306649<br>Anti-Prrx1 Origene Cat# TA803116,RRID:AB_2620166
Peak calling parameters	MACS2 peak caller tool with parameter -p 1e-5 for sharp peaks calling and with parameter --broad --broad-cutoff 0.1 for broad peaks calling. We obtained average 1755 peaks of above 5-fold enrichment based on the above peak calling parameters.

Data quality	We repeated experiments on different types of cells (human CAFs, active fibroblast of murine wound tissue, mouse fibroblasts) to ensure the accuracy of the results.
Software	Trimmomatic; Bowtie; samtools; Picard tools; MACS2; ROSE2; ChIPseeker.

Flow Cytometry

Plots

Confirm that:

- [x] The axis labels state the marker and fluorochrome used (e.g. CD4-FITC).
- [x] The axis scales are clearly visible. Include numbers along axes only for bottom left plot of group (a 'group' is an analysis of identical markers).
- [x] All plots are contour plots with outliers or pseudocolor plots.
- [x] A numerical value for number of cells or percentage (with statistics) is provided.

Methodology

Sample preparation	For cell death analysis, detached dead cells were collected within the culture supernatant prior to washing and trypsinization of remaining attached cells. Supernatant dead cells were aggregated with detached viable cells prior to staining with APC-conjugated Annexin-V (BD Biosciences, 550474) and PI.
Instrument	Flow cytometry analysis was performed on FACS Calibur (BD biosciences, CA). Sorting of total viable GFP(+) and GFP(-) cells was performed on FACSAria™ sorters (BD Biosciences).
Software	Data were acquired using either BD FACSDiva software (BD Biosciences).
Cell population abundance	For cell death analysis, Alive cells (Annexin-V and propidium iodide negative) are part of a pure population of CAF cells. For sorting of GFP(+)/(-) cells, Alive cells are sorted using 4-way purity mode to maximize purity. Contamination of sorted cell samples was minimal as determined by fluorescence microscope.
Gating strategy	For flow cytometry analysis, cells were gated by FSC area vs. SSC area, and singlets were gated by FSC area vs FSC height, followed by SSC area vs. SSC height. For cell death analysis, cells that were Annexin V-APC+ and PI+ were identified as dead cells. For sorting of GFP(+)/(-) cells, cells were gated by FSC area vs. SSC area, and singlets were gated by FSC area vs FSC height. GFP cells were subsequently gated by FITC vs. SSC area.

- [x] Tick this box to confirm that a figure exemplifying the gating strategy is provided in the Supplementary Information.
